# Temporal challenges in detecting balancing selection from population genomic data

**DOI:** 10.1093/g3journal/jkae069

**Published:** 2024-03-29

**Authors:** Vivak Soni, Jeffrey D Jensen

**Affiliations:** School of Life Sciences, Center for Evolution & Medicine, Arizona State University, Tempe, AZ 85281, USA; School of Life Sciences, Center for Evolution & Medicine, Arizona State University, Tempe, AZ 85281, USA

**Keywords:** balancing selection, distribution of fitness effects, demography, recombination rate, mutation rate, background selection

## Abstract

The role of balancing selection in maintaining genetic variation remains an open question in population genetics. Recent years have seen numerous studies identifying candidate loci potentially experiencing balancing selection, most predominantly in human populations. There are however numerous alternative evolutionary processes that may leave similar patterns of variation, thereby potentially confounding inference, and the expected signatures of balancing selection additionally change in a temporal fashion. Here we use forward-in-time simulations to quantify expected statistical power to detect balancing selection using both site frequency spectrum- and linkage disequilibrium-based methods under a variety of evolutionarily realistic null models. We find that whilst site frequency spectrum-based methods have little power immediately after a balanced mutation begins segregating, power increases with time since the introduction of the balanced allele. Conversely, linkage disequilibrium-based methods have considerable power whilst the allele is young, and power dissipates rapidly as the time since introduction increases. Taken together, this suggests that site frequency spectrum-based methods are most effective at detecting long-term balancing selection (>25*N* generations since the introduction of the balanced allele) whilst linkage disequilibrium-based methods are effective over much shorter timescales (<1*N* generations), thereby leaving a large time frame over which current methods have little power to detect the action of balancing selection. Finally, we investigate the extent to which alternative evolutionary processes may mimic these patterns, and demonstrate the need for caution in attempting to distinguish the signatures of balancing selection from those of both neutral processes (e.g. population structure and admixture) as well as of alternative selective processes (e.g. partial selective sweeps).

## Teaser

This study uses simulations to quantify the statistical power underlying common approaches to detect balancing selection. Soni and Jensen find that allele frequency-based methods are underpowered unless stable balancing selection is occurring for considerable timespans, whilst linkage disequilibrium-based patterns have power immediately upon the introduction of the balanced allele, though this power dissipates rapidly. Finally, the authors highlight various other processes that may mimic the signal of balancing selection, outlining current best practices for assessing the contributions of these various evolutionary forces.

## Introduction

The term balancing selection encapsulates a variety of evolutionary processes that maintain variability in populations (see review of Fijarczyk and Babik 2015), such that alleles may be held at appreciable frequencies over potentially considerable timescales (Lewontin 1987). Heterozygote advantage (also referred to as overdominance), frequency-dependent selection, and both spatially and temporally variable selection may all be placed in this category (Nielsen 2005).

Before direct quantifications of population-level genetic variation became feasible, a dominant debate within population genetics was centered upon whether levels of genetic variation were expected to be minimal or substantial. Selection was the dominant proposed mechanism shaping variation in what came to be known as the *classical/balanced* debate (Lewontin 1974; Crow 1987), be it purifying selection reducing genetic variation, or balancing selection maintaining variation (Dobzhansky 1955). Although the advent of next generation sequencing would yield molecular evidence demonstrating that levels of polymorphism were indeed plentiful, an alternative explanation became dominant over explanations invoking widespread balancing selection. Namely, the Neutral Theory of Molecular Evolution (Kimura 1968, 1983) posited that observed variation may simply be neutral alleles in the process of drifting to ultimate fixation or loss. This hypothesis has since been widely supported (Jensen *et al*. 2019).

Today, the role that balancing selection has in maintaining levels of variation remains unresolved (Gillespie 1991; Nielsen 2005), though evidence continues to suggest that it is generally a relatively minor player relative to what was once believed, and a small handful of gene-level examples have been described (e.g. Asthana *et al*. 2005; Andrés *et al*. 2009; Quintana-Murci and Clark 2013; and see reviews of Fijarczyk and Babik 2015; Bitarello *et al*. 2023). The most discussed examples of sites likely experiencing balancing selection include those involved in the major histocompatibility complex (MHC) in vertebrates (Kelley *et al*. 2005; Piertney and Oliver 2006; Spurgin and Richardson 2010), plant self-incompatibility (Lawrence 2000; Castric and Vekemans 2004; Goldberg *et al*. 2010), and sex determination (Charlesworth 2004). Where attempts have been made to quantify the number of sites experiencing balancing selection, estimates have naturally been limited to functional regions, with a high level of uncertainty (Bitarello *et al*. 2018; Soni *et al*. 2022). Apart from functional validation, recent years have seen a focus on utilizing whole-genome sequencing data to identify candidate loci potentially experiencing balancing selection, based on consistency with expected patterns of variation. These scans have frequently been performed on human populations, with balanced loci implicated in immune response (e.g. Andrés *et al*. 2009; Bitarello *et al*. 2018), including host-pathogen interactions (e.g. Leffler *et al.* 2013). Given that the signals of balancing selection are expected to change in a temporal fashion, these studies have harnessed different population genetic statistics depending on the time frame of interest (see below).

### Signatures of balancing selection

The temporal history of a balanced allele can be split into multiple phases, each with a unique signature. Fijarczyk and Babik (2015) categorized these signatures into recent (<0.4*N_e_* generations), intermediate (0.4–4*N_e_* generations), and ancient (>4*N_e_* generations) balancing selection, where *N_e_* is the effective population size, whilst Bitarello *et al*. (2023) characterized recent balancing selection as <10^6^ years old (<4*N_e_* generations in humans); long-term balancing selection as between 10^6^ and 7 × 10^6^ years old (between 4*N_e_* and 4*N_e_* + *T*_div_ generations in humans, where 4*N_e_* + *T*_div_ is the expected coalescence time between lineages that diverged *T*_div_ generations ago), and ultra-long-term balancing selection as greater than 7 × 10^6^ years old. Due to the differing terminology, mentions of timescales in this study are accompanied by the needed details. The initial phase is that of a partial selective sweep, whereby the allele in question increases in prevalence over time in the population until reaching the balanced frequency, conditional on escaping stochastic loss. These partial sweep patterns have been well-described in the literature, and may be expected to include, for example, an excess of intermediate frequency alleles as well as extended linkage disequilibrium (LD) and haplotype structure owing to the associated genetic hitchhiking effects (see reviews of Crisci *et al*. 2012; Charlesworth and Jensen 2021), and may limit population differentiation resulting in a weaker genetic structure at genes experiencing balancing selection (Schierup *et al*. 2000). Once achieving the balanced frequency, recombination will gradually shuffle these haplotype and LD patterns, and thus may in principle reduce detectability (Wiuf *et al*. 2004; Charlesworth 2006; Pavlidis *et al*. 2012). Finally, if balancing selection persists in species whose divergence time significantly predates the expected coalescent time for either species, a pattern of trans-species polymorphisms may become informative (Klein *et al*. 1998; Leffler *et al*. 2013).

Despite balancing selection leaving numerous signatures, a number of other processes may result in highly similar genomic patterns. For example, identifying trans-species polymorphisms can be fraught with difficulty, given that introgression, recurrent mutation, and incomplete lineage sorting can all confound inference (Figueroa *et al*. 1988). Likewise, the initial sweeping of the balanced mutation is indistinguishable from partial selective sweeps (i.e. a balanced allele increasing in frequency toward an intermediate equilibrium will initially generate the same pattern as a beneficial allele increasing in frequency toward fixation). Similarly, neutral demographic processes such as population bottlenecks may also result in such rapid increases in frequency (Jensen *et al*. 2007; Harris and Jensen 2020), and given that many commonly studied populations and species are thought to have experienced recent and severe bottlenecks [e.g. humans (Gutenkunst *et al*. 2009; Gravel *et al*. 2011; Excoffier *et al*. 2013) and *Drosophila melanogaster* (Li and Stephan 2006), as well as a variety of human pathogens (Irwin *et al*. 2016; Jensen 2021; Morales-Arce *et al*. 2021; Terbot, Cooper, *et al*. 2023)], failing to account for this population history is likely to result in mis-inference (Johri, Aquadro, *et al*. 2022; Howell *et al*. 2023; Terbot, Johri, *et al*. 2023).

The observed excess of intermediate frequency alleles that follows the initial sweeping phase may also be generated by population structure (Lewontin and Krakauer 1973; Beaumont 2005; Narum and Hess 2011). Although it is often argued that neutral demographic processes, including population size change and structure, can be distinguished from positive and balancing selection given that the former have genome-wide effects whilst the latter are limited to selected and neighboring loci, it has been well demonstrated that unaccounted for demographic processes may greatly increase false-positive rates when performing such genomic scans (Teshima *et al*. 2006; Thornton and Jensen 2007; Jensen *et al*. 2008; Soni *et al*. 2023), whilst unaccounted for recombination and mutation rate heterogeneity can similarly result in mis-inference by generating localized peaks or troughs of diversity (Soni *et al*. 2023, 2024). In this light, it is notable that the majority of balancing selection studies to date neglect these various contributing factors to some degree, and have employed genome-wide scans using a number of either individual or compound statistics (e.g. Andrés *et al*. 2009; Bitarello *et al*. 2018) or likelihood ratio comparisons (e.g. DeGiorgio *et al*. 2014; Cheng and DeGiorgio 2020).

Hence, to account for these constantly acting evolutionary processes—including purifying and background selection (BGS) effects, genetic drift as partially modulated by population history, as well as mutation and recombination rate heterogeneity—Johri, Eyre-Walker, *et al*. (2022), Johri, Aquadro, *et al*. (2022) recently proposed the necessity of constructing an appropriate evolutionary baseline model incorporating these processes prior to scanning for the effects of comparatively rare and episodic processes such as balancing or positive selection. Namely, given that multiple processes have overlapping predictions in terms of expected levels and patterns of variation, such that, for example, selective effects may often be confused with and confounded by demographic effects (e.g. Charlesworth *et al*. 1993; Charlesworth 1996, 2013; Jensen *et al*. 2005; Kaiser and Charlesworth 2009; O’Fallon *et al*. 2010; Nicolaisen and Desai 2013; Zeng 2013; Ewing and Jensen 2016; Johri *et al*. 2021; Charlesworth and Jensen 2022; Soni *et al*. 2023), an appropriate baseline model of this sort is necessary to reduce false-positive rates, and importantly to quantify the identifiability of these rare and episodic processes within this more realistic context.

Despite the renewed interest in balancing selection in recent years, there has yet to be a systematic attempt to quantify how much inference power exists at various time scales, once accounting for these alternative evolutionary processes. We thus utilized simulations to assess the effectiveness of a common composite likelihood ratio (CLR)-based method, *B*_2_ (Cheng and DeGiorgio 2020), as well as a common linkage-based method, *iHS* (Voight *et al*. 2006), for detecting balancing selection under a variety of evolutionary baseline models. In brief, the *B*_2_ method is one of a number of *B* statistics that utilize a mixture model combining the expectation of the site frequency spectrum (SFS) under neutrality and under balancing selection to infer the expected SFS at both a putatively selected site and at increasing distances away from that site (Cheng and DeGiorgio 2020). The *B*_2_ statistic specifically incorporates both the full unfolded SFS as well as substitution data. The *iHS* statistic is an extension of the extended haplotype homozygosity (*EHH*; Sabeti *et al*. 2002) statistic. *EHH* is defined by the probability that 2 randomly chosen chromosomes carrying a core haplotype of interest are identical by descent for the entire interval from the core region to the target locus. The integrated haplotype score (*iHS*) compares the integral of the EHH curve for ancestral and derived alleles, with the expectation that the EHH curve will be greater under selection than under neutrality (Voight *et al*. 2006). These examined statistics thus span the primary expected SFS- and LD-based intra-population signals of balancing selection.

Our results suggest that *iHS* and *B*_2_ are useful approaches for detecting balancing selection over extremely short (<1*N*_ancestral_ generations) and extremely long (>25*N*_ancestral_ generations) timescales—leaving a considerable time frame in which inference power is minimal. We also demonstrate that population structure, admixture, and partial sweeps can all mimic these detected signals of balancing selection within certain parameter ranges, but importantly that via baseline model construction this lack of identifiability may be quantified rather than simply resulting in high false-positive rates.

## Materials and methods

### Simulations

A single population was simulated using forward-in-time software SLiM 4.0.1 (Haller and Messer 2023). Two chromosomal structures were simulated: (1) a strictly neutral background model consisting of a single 50 kb region, and (2) a model that incorporates a DFE in functional regions. For this second model, the numbers of introns and exons per functional region were obtained from Sakharkar *et al*. (2004), with mean intron length taken from Hubé and Francastel (2015). Finally, the lengths of exons and intergenic regions were averages estimated from Ensembl's GRCh38.p14 dataset (1000 Genomes Project Consortium 2015), obtained from Ensembl release 107 (Cunningham *et al*. 2022), and were used to simulate a chromosome with human-type structure and parameterizations. Specifically, each replicate was made up of 3 functional regions separated by intergenic regions of size 4,322 bp. Each functional region contained 9 exons (of size 1,317 bp) and 8 introns (of size 1,520 bp), resulting in a functional region length of 24,013 bp. The total chromosome length was 85,005 bp (see Supplementary Fig. 1 for a schematic representation of the structure of the functional region).

Mutations in intronic and intergenic regions were all strictly neutral, whilst exonic mutations were drawn from a DFE comprised of 4 fixed classes (following Johri *et al*. 2020), with frequencies denoted by *f_i_*: *f*_0_ with 0 ≤ 2*N*_ancestral_  *s* < 1 (i.e. effectively neutral mutations), *f*_1_ with *1* ≤ 2*N*_ancestral_  *s* < 10 (i.e. weakly deleterious mutations), *f*_2_ with 10≤ 2*N*_ancestral_  *s* < 100 (i.e. moderately deleterious mutations), and *f*_3_ with 100 ≤ 2*N*_ancestral_  *s* (i.e. strongly deleterious mutations), where *N*_ancestral_ is the initial population size and *s* is the reduction in fitness of the mutant homozygote relative to the wild-type. All mutations were semi-dominant (*h* = 0.5) and, within each bin, *s* was drawn from a uniform distribution. Every third site within exons was simulated to be neutral (i.e. to serve as synonymous sites). Overall, 6 different discrete DFEs were simulated.

The ancestral population size (*N*_ancestral_) was set at 10,000, the estimated effective population size (*N_e_*) in humans (Takahata 1993). Due to this modest population size, no scaling of simulation parameters was necessary. The fixed mutation rate, *μ*, was set at 2.5 × 10^−8^ per base per generation (Nachman and Crowell 2000), and the fixed recombination rate, *r*, was 1 × 10^−8^ per base per generation (Payseur and Nachman 2000); variable rate simulations are discussed in the sections below. A single balanced mutation was introduced at the center of the simulated chromosome (position 25,000 in neutral background simulations; position 40,342 in functional region simulations) immediately after the 10*N*_ancestral_ generation burn-in period. A total of 200 replicates were simulated for each simulation scenario, with 100 chromosomes sampled at 0.01*N*, 0.1*N*, 1*N*, 10*N*, 25*N*, 50*N*, 75*N*, and 100*N* generations since the introduction of the balanced mutation for the neutral background simulations.

### Modeling balancing selection

The balanced mutation was modeled under negative frequency-dependent selection, with a dominance coefficient, *h* = 0.5, and an equilibrium frequency of 0.5. Simulation replicates in which the balanced mutation failed to establish (i.e. reach a frequency of 0.1) were discarded. Negative frequency-dependent selection was modeled such that the selection coefficient of the balanced mutation was dependent on its frequency in the population:


$$S_{\mathrm{bp}}=F_{\mathrm{eq}}\text{–}F_{\mathrm{bp}}$$

where *S*_bp_ is the selection coefficient of the balanced mutation, *F*_eq_ is the equilibrium frequency of the balanced mutation, and *F*_bp_ is the frequency of the balanced mutation.

### Simulating population size change and variable mutation and recombination rates

Four scenarios were simulated: (1) fixed mutation rate and fixed recombination rate, (2) fixed mutation rate and variable recombination rate, (3) variable mutation rate and fixed recombination rate, as well as (4) variable mutation rate and variable recombination rate, with each scenario simulated in the presence of selection. Where rates were variable, each 1 kb region of the simulated chromosome had a different rate. Rates were drawn from a uniform distribution such that the chromosome-wide average was approximately the fixed rate. For variable recombination rates, the minimum and maximum parameters of the uniform distribution were 0.124 and 4.930 cM/Mb, respectively; the maximum value is that of the sex-averaged Kong *et al*. (2010) human recombination map. For variable mutation rates, the minimum and maximum parameters of the uniform distribution were set at 1.3 × 10^−8^ and 3.4 × 10^−8^ per site per generation, to give a mean rate across each replicate that was equal to the fixed rate.

Four population histories were simulated: (1) demographic equilibrium, (2) instantaneous 100% population expansion, (3) instantaneous 50% population contraction, and (4) instantaneous 99% population contraction. In each case, 2 sets of simulations were run: population size change occurring 74*N*_ancestral_ generations after the introduction of the balanced mutation (i.e. 1*N* generations before sampling), and separately, population size change occurring immediately as the balanced mutation was introduced. This was to ensure that population size change was recent enough to impact both the *B*_2_ and *iHS* methods. See Supplementary Fig. 2 for visual representation of population histories.

### Simulating human demographic models

Two published models of human demographic history were additionally simulated: (1) Gravel *et al*. (2011) and (2) Hu *et al*. (2023). The former was simulated with no modification, whilst the latter was simulated with the initial population expanding [to account for the difference between the human ancestral population size (27,716, taken from Schrago 2014) and the population size at the start of the Hu *et al*. model prior to the population bottleneck (93,619)]. For the Gravel *et al*. (2011) model, 100 chromosomes were sampled from each of the 3 simulated populations. For the Hu *et al*. (2023) model, 100 chromosomes were sampled from the single simulated population. A discrete DFE was modeled using the parameterizations from Johri *et al*. (2023). All other parameterizations were as simulated in the above scenarios. The Gravel *et al*. (2011) model was also simulated in the absence of balancing selection in order to obtain false-positive rates, whilst the Hu *et al*. (2023) model was also simulated using a number of equilibrium frequencies of the balanced mutation (*f*_eq_ = 0.1, 0.25, 0.5, and 0.75) to evaluate how inference power was impacted by equilibrium frequency.

### Partial sweep simulations

To simulate partial selective sweeps, the neutral background simulations were run as described above. The mutation experiencing positive selection was simulated with the same parameterizations as the balanced mutation, without the addition of frequency-dependent selection. One hundred chromosomes were sampled when the positively selected mutation reached a frequency of 0.5.

### Stochastic loss simulations

Neutral background and functional region simulations were run as above, with a mutation experiencing positive selection introduced (as per the partial sweep simulations). The simulation was terminated once the positively selected mutation either established (i.e. reached a frequency of 0.1) or was lost, with a record kept of the number of lost and established replicates. These simulations were run at population-scaled beneficial selection coefficients of 2*N_e_s* = 100 and 1,000. Ten thousand replicates were simulated for each scenario.

### Population structure and admixture simulations

Coalescent simulations were run in fastsimcoal2 (Excoffier *et al*. 2013). One thousand replicates were run for each scenario. A 2-population simulation was run in which 2 populations of size *N* = 10,000 were simulated under a number of population-scaled migration rates: *Nm* = 0, 0.01, 0.05, 0.1, or 0.2. Migration was symmetric between the populations. One hundred chromosomes were sampled from 1 population.

Admixture simulations were run in which a single ancestral population split into 2 daughter populations (all of size *N* = 10,000) after 10*N* generations, with strong migration (*Nm* = 0.25) persisting between the daughter populations for 5*N* generations, followed by 5*N* generations of isolation. One hundred chromosomes were sampled from 1 population.

Hidden structure simulations were run in which a single ancestral population split into 2 daughter populations (all of size *N* = 10,000) after 10*N* generations. Fifty chromosomes were sampled from each daughter population.

### Detecting balancing selection using *B*_2_

The *B*_2_ method was run on each simulated replicate to detect balancing selection, as implemented in the BalLeRMix+ software (Cheng and DeGiorgio 2020). Allele frequency input files were generated for each simulation replicate using a custom python script, whilst SFS files were generated via BalLeRMix+. Inference was performed at each single nucleotide polymorphism (SNP), with default parameters. Execution was via the following command line:

python3 BalLeRMix+_v1.py -i inputFile --spect sfsFile -o outputFile --usePhysPos --rec 1e-8.

### Detecting balancing selection using *iHS*

The *iHS* method was run on each simulated replicate to detect balancing selection, as implemented in the SelScan 2.0 software (Szpiech 2021). Input .hap and .map files were generated using a custom python script. Inference was performed at each SNP. Execution was via the following command line:

Selscan --ihs --hap hapFile --map mapFile --out outPath --trunc-ok --keep-low-freq.

### Generating receiver operating characteristic (ROC) curves

True-positive rates (TPRs) and false-positive rates (FPRs) were calculated across 100 bp nonoverlapping windows. If a SNP was within 50 bp of the balanced mutation, and the inference threshold was met, then the window containing that SNP was defined as a true-positive. For coalescent simulations, the FPR was calculated for 100 evenly distributed thresholds between 0 and the maximum *B*_2_ or *iHS* value across all simulation replicates.

### Calculating summary statistics

Summary statistics were calculated across 2 kb sliding windows with a step size of 1 kb, using the python implementation of libsequence (version 1.8.3) (Thornton 2003).

## Results and discussion

Simulations were run in SLiM4.0.1 (Haller and Messer 2023) in order to quantify power to detect balancing selection using the SFS-based composite likelihood method *B*_2_ (Cheng and DeGiorgio 2020), as well as the linkage-based method *iHS* (Voight *et al*. 2006), with inference performed at each SNP. Parameterizations for these simulations were taken from the human population genetic literature (see *Materials and methods* for details). Beginning with the simplest model of an equilibrium population, a neutral genetic background, and fixed mutation and recombination rates, we sequentially modeled increasingly complex and realistic null models ultimately consisting of nonequilibrium population histories, full distributions of fitness effects, and heterogenous mutation and recombination rates, in order to study the factors dictating the underlying statistical power to detect the action of balancing selection. In each case, balancing selection was modeled as negative frequency-dependent selection acting on a single balanced allele. This model was chosen as the overall dynamics should be relevant to many forms of balancing selection.

### Equilibrium, neutral genetic background, fixed rate model

**Fig. 1.**
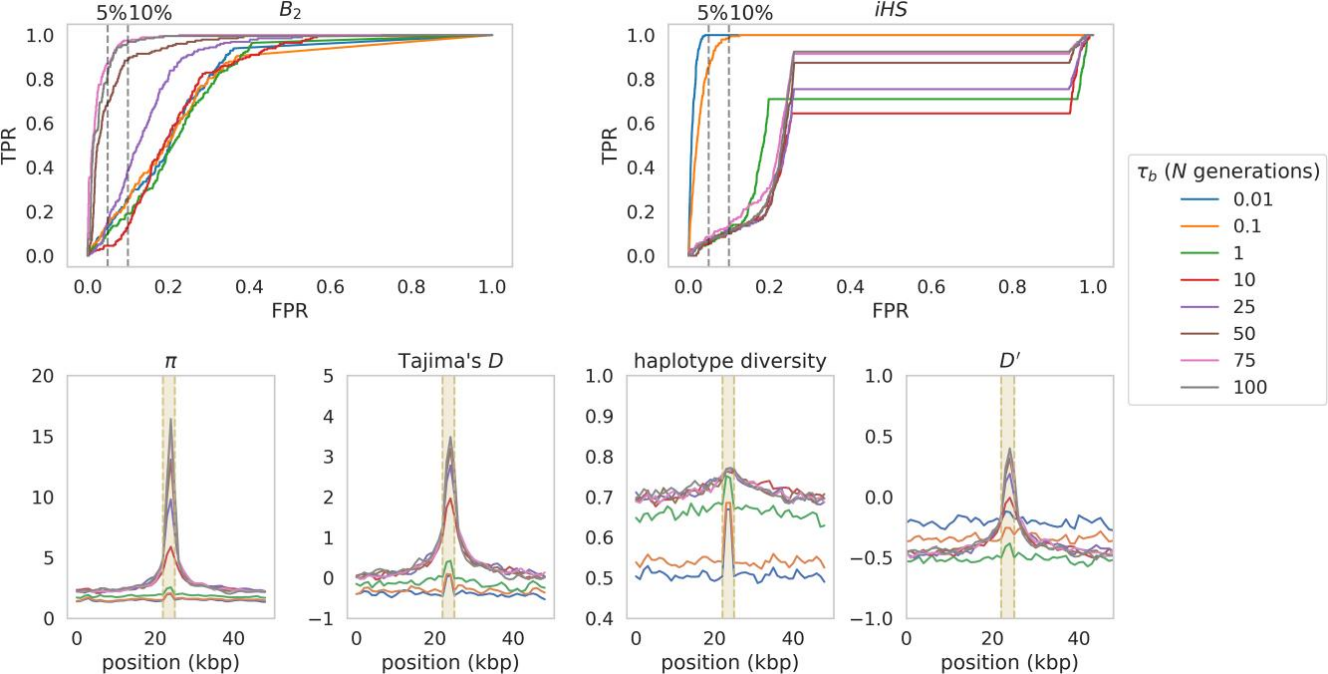
Top row presents ROC curves, providing the change in true-positive rate (TPR) as the false-positive rate (FPR) increases, for balancing selection inference in an equilibrium population with fixed mutation and recombination rates on a neutral genetic background, across 200 simulated replicates, using the *B*_2_ approach (top left) and the *iHS* statistic (top right). The simulated population was sampled at a number of values of *τ_b_* (time since the introduction of the balanced allele). *iHS* and *B*_2_ inference were performed at each SNP, and ROC curves were generated using 100 bp windows. Dashed vertical lines provide the 5% and 10% FPR thresholds, respectively. The bottom row provides the corresponding summary statistics, estimated across 2 kb sliding windows with a step size of 1 kb. From bottom left to bottom right: nucleotide diversity, Tajima's *D*, haplotype diversity, and mean *D*′. Shaded regions indicate the window in which the balanced mutation is segregating.

The simplest simulated scenario was one in which only neutral mutations were simulated in an equilibrium population, under fixed mutation and recombination rates, with a balanced mutation introduced in the center of the genomic region. Inference results are shown in Fig. 1. The *iHS* statistic had considerable power immediately after the introduction of the balanced mutation, though power rapidly dissipated as the time since introduction (*τ_b_*) of the balanced mutation increased (to the extent that there existed little power to detect anything beyond 0.01*N* generations). As discussed above, a balanced mutation escaping stochastic loss initially takes the trajectory of a partial selective sweep upon introduction into a population, resulting in extended LD as can be seen by the initial region-wide elevation of *D*′ (Fig. 1), which measures the scaled coefficient of linkage (Lewontin 1964). This region-wide pattern dissipates rapidly as time since *τ_b_* increases. Once the balanced mutation reaches equilibrium frequency however, LD increases around the balanced mutation, though at a much shorter distance (dictated by the recombination rate) as it fluctuates about the equilibrium frequency. Concurrently, balancing selection initially generates a greater number of differentiated haplotypes, and as *τ_b_* increases, recombination breaks up these haplotypes, a pattern borne out in levels of haplotype diversity (Fig. 1) initially increasing around the balanced mutation.

Thus, as extended LD and the skew in haplotype diversity around the balanced mutation dissipated, so did inference power with *iHS*, which is dependent on a slower decay of identity by descent under selection than under neutrality (Voight *et al*. 2006), and therefore even at *τ_b_* = 1*N* the *iHS* statistic had essentially no power to detect balancing selection. This is consistent with Sabeti *et al*.'s (2002) suggestion that selective events less than 400 generations old (i.e. 0.04*N* generations) should leave a detectable long-range LD signature. This suggests that one of the limitations of using linkage-based methods for detecting balancing selection is the inability to distinguish partial selective sweeps from the initial sweeping phase of a newly introduced balanced mutation. In order to explore this confounding effect, partial sweep simulations were run under two different selection regimes (2*N_e_s* = 100 or 1,000), sampling when the introduced beneficial mutation reached a frequency of 0.5, and performing inference with the *iHS* statistic (see *Materials and methods* for further details). Supplementary Fig. 3 compares receiver operating characteristic (ROC) plots for partial sweeps with balancing selection inference at *τ_b_* = 0.01*N*, as well as haplotype diversity and mean *D*′ for these simulations. Because the initial trajectory of the balanced mutation mirrors that of the partial sweep, disentangling the two processes is not possible from *iHS* inference alone. Indeed, the patterns of haplotype diversity were essentially identical as well, with levels slightly elevated under balancing selection, likely due to the much higher strength of selection acting on the balanced mutation upon its introduction into the population (the strength of selection acting on the balanced mutation, *s*_bp_, was such that *s*_bp_ = *f*_eq_ − *f*_bp_ where *f*_eq_ was the equilibrium frequency of the balanced mutation, here 0.5, and *f*_bp_ was the frequency of the balanced mutation in the population). This increased selection intensity also resulted in the higher levels of extended LD (as measured by mean *D*′). Although the focus here is on single time point data (i.e. the most common type of data used for population genomic inference), having access to data sampled at multiple time points would facilitate the tracking of allele frequency trajectories, potentially improving inference (Fijarczyk and Babik 2015).

By contrast, the *B*_2_ method had little power to detect recent balancing selection (Fig. 1), but much greater power over extremely long timescales (*τ_b_* > 25*N*). As *τ_b_* increased, new mutations arose on the balanced haplotype, as evidenced by the resultant increase in both *π* and Tajima's *D* (Tajima 1989; Fig. 1, and see Charlesworth and Jensen 2023) around the balanced locus. At shorter timescales the skew toward intermediate frequency alleles was limited and consequently there was little power to detect balancing selection with the SFS-based *B*_2_ approach.

Because of the limited power beyond *τ_b_* = 0.1*N* with the *iHS* statistic and below *τ_b_* = 25*N* with the *B*_2_ method, further analyses were limited to performing balancing selection inference using at *τ_b_* = 0.01*N* and 0.1*N* with the *iHS* statistic, and *τ_b_* = 25*N*, 50*N*, and 75*N* using the *B*_2_ method.

### Balancing selection inference in the presence of purifying and background selection

**Fig. 2.**
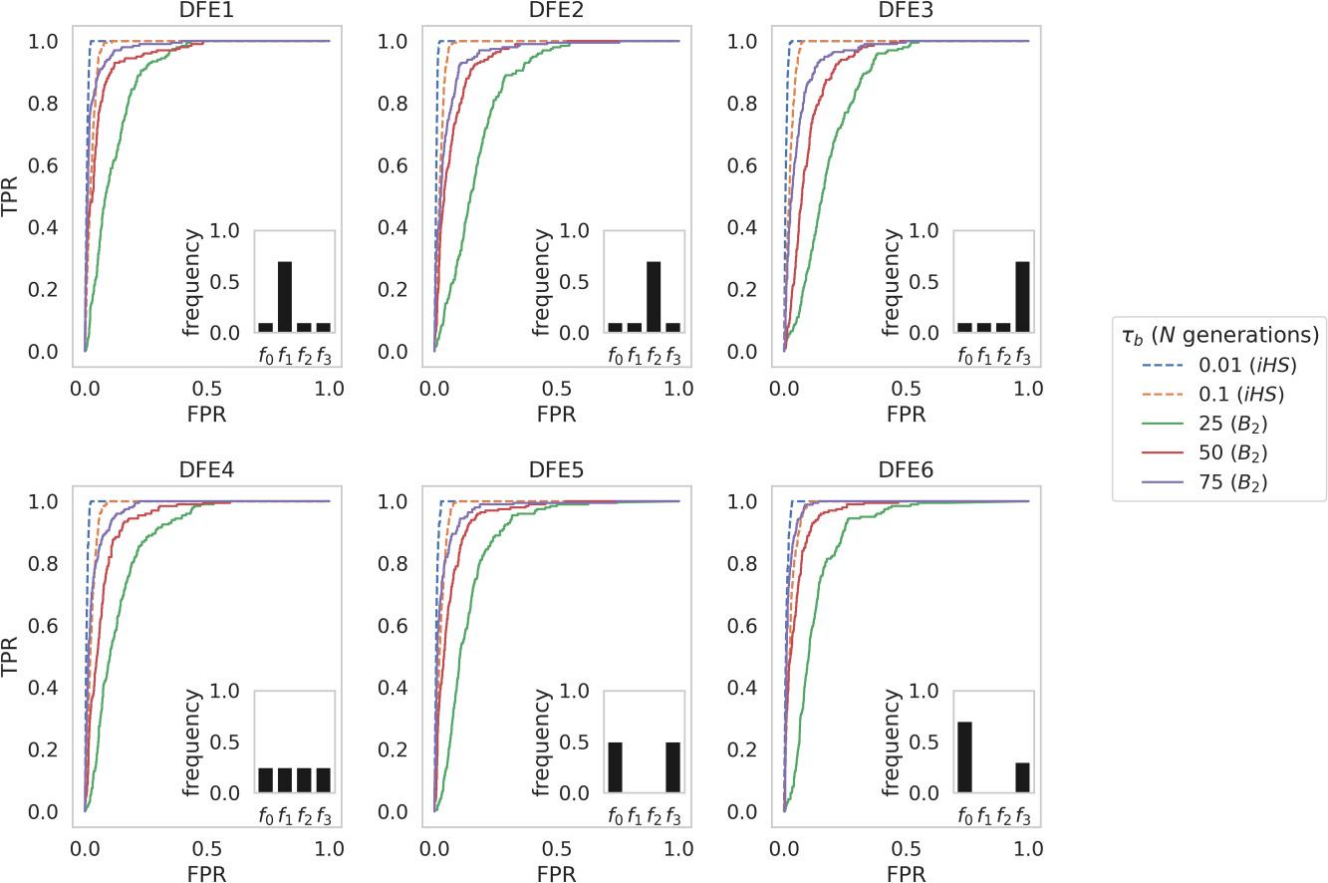
ROC curves presenting the change in true-positive rate (TPR) as the false-positive rate (FPR) increases, for balancing selection inference in an equilibrium population across 6 different DFEs, with fixed mutation and recombination rates across 200 simulated replicates, using the *B*_2_ approach and the *iHS* statistic. Timescales for inference were limited to those in which each method performed best: 0.01*N* and 0.1*N* generations since the introduction of the balanced mutation for *iHS*, and 25*N*, 50*N*, and 75*N* generations since introduction for *B*_2_. Inference was performed at each SNP, and ROC curves were generated using 100 bp windows. Inlayed plots provide the discrete DFE used for each panel. Exonic mutations were drawn from a DFE comprised of 4 fixed classes (following Johri *et al*. 2020), whose frequencies were denoted by *f_i_*: *f_0_* with 0 ≤ 2*N*_ancestral_  *s* < 1 (i.e. effectively neutral mutations), *f*_1_ with 1 ≤ 2*N*_ancestral_  *s* < 10 (i.e. weakly deleterious mutations), *f*_2_ with 10 ≤ 2*N*_ancestral_  *s* < 100 (i.e. moderately deleterious mutations), and *f*_3_ with 100 ≤ 2*N*_ancestral_  *s* (i.e. strongly deleterious mutations), where *N*_ancestral_ was the initial population size and *s* the reduction in fitness of the mutant homozygote relative to wild-type.

To incorporate the effects of purifying and background selection, functional elements were simulated, each separated by intergenic regions (see *Materials and methods* and Supplementary Fig. 2 for simulation details and demographic history schematics, respectively), with intergenic and intronic regions evolving neutrally. Mutations within exonic regions were draw from a discrete DFE, with 6 separate DFEs evaluated (following Johri *et al*. 2020) as depicted in Fig. 2. The 6 DFEs included: (1) an excess of weakly deleterious mutations; (2) an excess of moderately deleterious mutations; (3) an excess of strongly deleterious mutations; (4) an equal distribution of selection coefficients; as well as (5) and (6) bimodal distributions, a DFE shape commonly inferred in empirical/experimental DFE estimates for newly arising mutations [i.e. via directed mutagenesis studies (e.g. Hietpas *et al.* 2011) or the statistical analysis of natural population data (e.g. Johri *et al.* 2020)]. These enabled us to evaluate balancing selection inference power under numerous purifying and background selection regimes.

The effects of purifying and background selection generally had little effect on power to detect balancing selection with either *iHS* or *B*_2_ (Fig. 2), as expected. The strength of selection acting on the balanced mutation when it was introduced into the population was so great that the partial sweep phase was relatively unaffected by interference from deleterious variation, and therefore *iHS* power to detect balancing selection in this early phase remained high. At higher values of *τ_b_* there was a small reduction in *B*_2_ inference power from DFEs 1–3 (i.e. as the excess of deleterious mutation moved from slightly to strongly deleterious), likely due to strongly deleterious variation landing on the balanced haplotype [as evidenced by a small reduction in *π* and Tajima's *D* (Tajima 1989) in DFE3 (Supplementary Fig. 4)].

Although these results were encouraging in terms of the minimal bias generated by purifying and background selection on balancing selection inference, it is notable that only simulations in which the balanced mutation established were considered. Further simulations were run in which the proportion of beneficial mutations that escaped stochastic loss (defined as reaching an establishment frequency of 0.1) were counted across 10,000 replicates, in order to evaluate the effects of purifying and background selection on the probability of a new beneficial/balanced mutation establishing in a population (Table 1; see *Materials and methods* for simulation details). The presence of purifying and background selection reduced the probability of escaping stochastic loss by approximately 10-fold (a reduction from roughly 0.1 under a neutral background to 0.01 on average across the 6 simulated DFEs) at 2*N_e_s* = 100. At 2*N_e_s* = 1,000, the probability of escaping stochastic loss was reduced from 0.58 on a neutral background to 0.1 on average across the 6 DFEs. It was notable that the lowest probability of escaping stochastic loss was under DFE3, in which an excess of strongly deleterious mutations were segregating. These results highlight that although purifying and background selection appeared to have little effect on balancing selection inference relative to a neutral background conditional on establishment, the presence of deleterious mutations drastically reduced the probability that these mutations will escape stochastic loss owing to the resulting Hill–Robertson interference (Hill and Robertson 1966).

**Table 1.** Proportion of beneficial mutations that escape stochastic loss under different background conditions and strengths of selection.

Background	2*N_e_s*	Proportion of beneficial mutations escaping stochastic loss	DFE probability/neutral probability
Neutral	100	0.0959	n/a
Neutral	1,000	0.5834	n/a
DFE1	100	0.0117	0.1220
DFE1	1,000	0.0948	0.1625
DFE2	100	0.0119	0.1241
DFE2	1,000	0.0868	0.1488
DFE3	100	0.0076	0.0792
DFE3	1,000	0.0951	0.1630
DFE4	100	0.0102	0.1064
DFE4	1,000	0.0913	0.1565
DFE5	100	0.0086	0.0897
DFE5	1,000	0.0935	0.1603
DFE6	100	0.0097	0.1011
DFE6	1,000	0.0913	0.1565

Escape from stochastic loss is defined as achieving a frequency of 0.1. See *Materials and methods* section for simulation details.

### Balancing selection inference in the presence of purifying and background selection under nonequilibrium population histories

Though the confounding effects of demography on the inference of selective sweeps have been well documented (Barton 2000; Kim and Nielsen 2004; Jensen *et al*. 2005, 2007; Nielsen 2005; Pavlidis *et al*. 2008; Pavlidis *et al*. 2010; Poh *et al*. 2014; Soni *et al*. 2023; see also reviews of Pavlidis and Alachiotis 2017; Stephan 2019), the effects of population history on balancing selection inference have been studied only in specific contexts (e.g. Bitarello *et al*. 2018; Cheng and DeGiorgio 2020; Soni *et al*. 2022). To date, there has been no systematic study of the effects of demography on the inference of balancing selection, particularly when purifying and background selection are also modeled via a realistic DFE. We simulated several instantaneous population size changes, in which *N*_current_ = 2, 0.5, or 0.01*N*_ancestral_ under 6 DFE models, where *N*_current_ was the population size at time of sampling, and *N*_ancestral_ was the population size prior to the size change. Simulations were run separately for *iHS* inference (in which the population size change occurred at the same time as the introduction of the balanced mutation, and sampling occurred 0.01*N*_ancestral_ generations later) and for *B*_2_ inference (in which the population size change occurred 1*N*_current_ generations prior to sampling, and sampling occurred 75*N*_ancestral_ generations since the introduction of the balanced mutation).

**Fig. 3.**
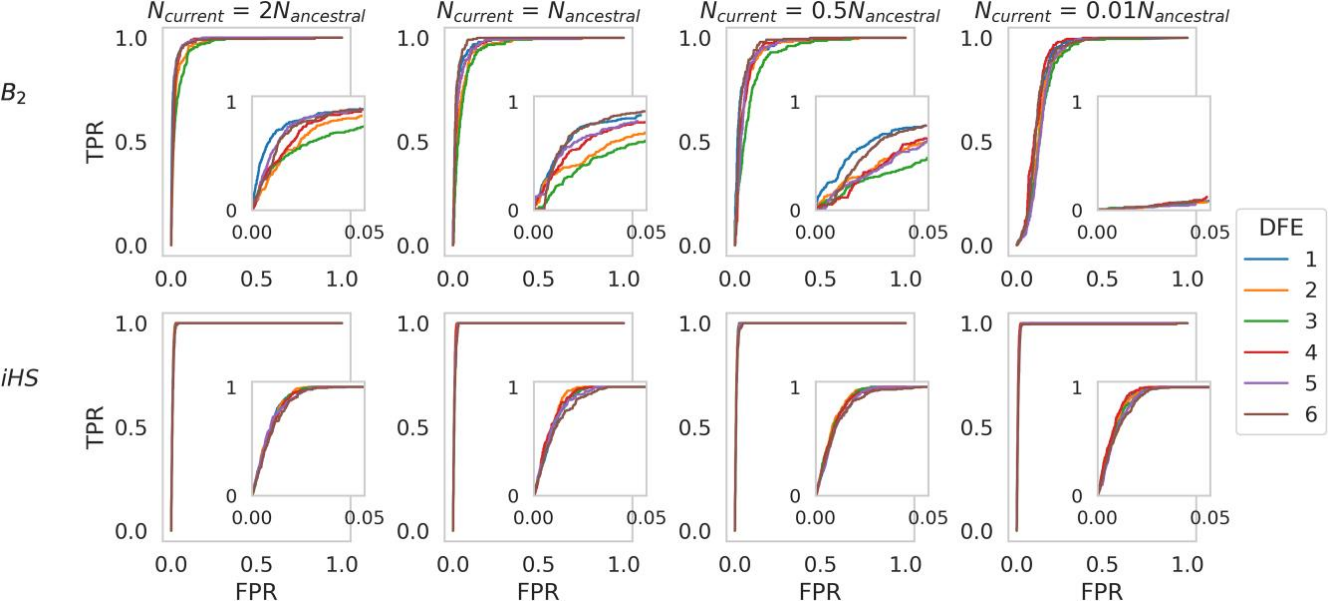
ROC curves presenting the change in true-positive rate (TPR) as the false-positive rate (FPR) increases, for balancing selection inference under four demographic histories across six DFEs, with fixed mutation and recombination rates across 200 simulated replicates, using the *B*_2_ approach and the *iHS* statistic. In each case, population size change was instantaneous. Top row: *B*_2_ inference results for simulations in which population size change occurred *N*_current_ generations before sampling, where *N*_current_ was the population size at time of sampling. Sampling occurred 75*N*_ancestral_ generations after the introduction of the balanced mutation, where *N*_ancestral_ was the initial population size. Bottom row: *iHS* inference results for simulations in which the population size change occurs 0.01*N*_ancestral_ generations before sampling (the same time as the introduction of the balanced mutation). Inset plots show zoomed in ROC curves covering FPR values from 0 to 0.05. *iHS* and *B*_2_ inferences were performed at each SNP, and ROC curves were generated using 100 bp windows. Exonic mutations were drawn from a DFE comprised of 4 fixed classes (following Johri *et al*. 2020), whose frequencies were denoted by *f_i_: f*_0_ with 0 ≤ 2*N*_ancestral_  *s* < 1 (i.e. effectively neutral mutations), *f*_1_ with 1 ≤ 2*N*_ancestral_  *s* < 10 (i.e. weakly deleterious mutations), *f*_2_ with 10 ≤ 2*N*_ancestral_  *s* < 100 (i.e. moderately deleterious mutations), and *f*_3_ with 100 ≤ 2*N*_ancestral_  *s* (i.e. strongly deleterious mutations), where *s* was the reduction in fitness of the mutant homozygote relative to wild-type. See Fig. 2 for plots of DFEs.

The *iHS* method proved relatively robust to population size change (Fig. 3) after 0.01*N*_ancestral_ generations since the introduction of the balanced mutation, regardless of the underlying DFE. Under the model of negative frequency-dependent selection, the strength of selection acting on the balanced allele at extremely low frequencies would be such that the partial sweep trajectory was not strongly impacted by fluctuations in population size (see *Materials and methods* for more details). As Supplementary Fig. 5 shows, summary statistics—most notably haplotype diversity and *D*′—were largely unperturbed by population size change, when conditioning on reaching the balanced frequency.

*B*_2_ inference was more revealing (Fig. 3), with power to infer balancing selection reduced under population contraction. Under the more severe contraction model (99% population size reduction) the resulting loss of power was greater, suggesting that the severity of the loss in power is a function of the severity of the population contraction. Indeed, population expansion (in which the population size doubled) resulted in a small increase in power due to the skew in the SFS toward rare alleles [supported by the genome-wide negative Tajima's *D* (Tajima 1989); Supplementary Fig. 6], thereby increasing the differentiation between the underlying genome-wide SFS and the specific locus experiencing balancing selection. Population contractions resulted in the reverse effect, as well as many segregating variants being fixed or lost, which can break up the balanced haplotype, thereby reducing power. Population bottlenecks also inflate the variance of the SFS and LD across the genome (Thornton and Jensen 2007; most notably with *D*′, as shown in Supplementary Fig. 6); identifying locus-specific selection patterns is therefore more challenging, as the underlying neutral distributions of these statistics becomes highly dispersed.

### The effects of variable recombination and mutation rates on balancing selection inference

Heterogeneity across the genome in both the rate of recombination (Kong *et al*. 2010; Cox *et al*. 2009; Rockman and Kruglyak 2009; Comeron *et al*. 2012; Kawakami *et al*. 2014; and see the review of Stapley *et al*. 2017) and the rate of mutation (Lynch 2010; Hodgkinson and Eyre-Walker 2011; Rahbari *et al*. 2016; Carlson *et al*. 2018; Pfeifer 2020; and see the review of Baer *et al*. 2007) has been observed across the genomes of numerous taxa. To assess the effects of variable mutation and recombination rates on balancing selection inference, simulated data was generated in which each 1 kb region had a rate drawn from a uniform distribution, such that the mean rate across each variable rate simulation replicate was equal to the fixed rate (see *Materials and methods* for further details). Three scenarios were simulated and compared to the fixed mutation and recombination rate inference results discussed above: fixed recombination/variable mutation rate; variable recombination/fixed mutation rate; and variable recombination/variable mutation rate.

**Fig. 4.**
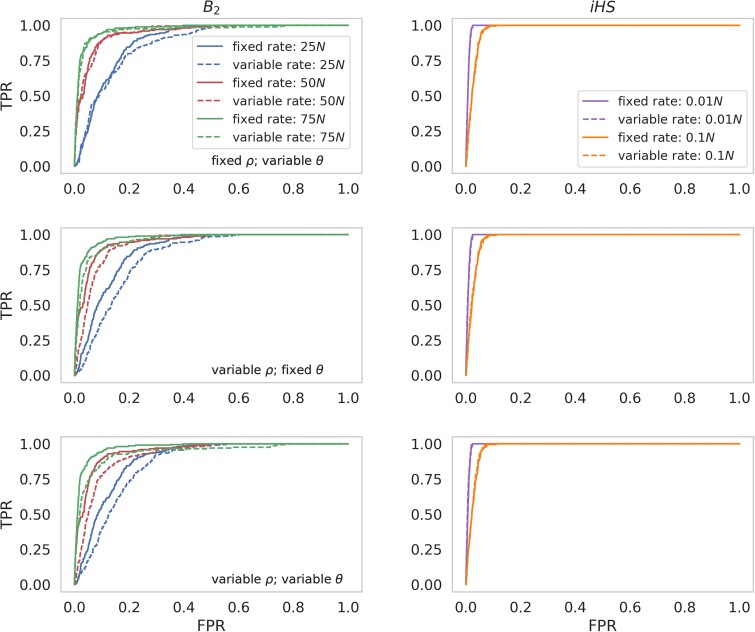
ROC curves presenting the change in true-positive rate (TPR) as the false-positive rate (FPR) increases, for balancing selection inference in an equilibrium population for DFE1 (see Fig. 2 for barplot of DFE1), comparing fixed mutation and recombination rates (solid line) with variable rates (dashed line) across 200 simulated replicates, using the *B*_2_ approach (left) and the *iHS* statistic (right). For variable rates, each 1 kb region had a rate drawn from a uniform distribution such that each simulated replicate has the same mean rate as the fixed rate comparison (see *Materials and methods* for further details). Inference was performed at each SNP, and ROC curves were generated using 100 bp windows. Timescales for inference were limited to those in which each method performed best: 0.01*N* and 0.1*N* generations since the introduction of the balanced mutation for *iHS*, and 25*N*, 50*N*, and 75*N* generations for *B*_2_.

Figure 4 compares inference power between variable and fixed rates for equilibrium population simulations under DFE1 (for results under the other five DFEs, see Supplementary Figs. 7–12, and see Supplementary Figs. 13–15 for the underlying summary statistics). The *iHS* statistic was generally robust to variable rates, likely owing again to the extremely strong selection acting on the balanced mutation immediately upon introduction. *B*_2_ inference was robust to mutation rate variation, with little change in power. At high values of *τ_b_*, the balanced haplotype will already be large enough to maintain power, even when the input of new mutations is reduced (i.e. in low mutation rate regions). By contrast, variable recombination rates resulted in a notable loss of power at all three values of *τ_b_* (25*N*, 50*N*, and *75N*), as regions with high recombination rates are likely to break up the balanced haplotype and thereby reduce the signature of balancing selection. It is notable that the extent of the reduction in power depended on both the time since introduction of the balanced mutation, and the underlying DFE (Supplementary Fig. 9). Variable rate simulations were also run under the three models of population size change (see Supplementary Fig. 2 for schematics of demographic models), with patterns largely mirroring those discussed in the preceding section (see Supplementary Figs. 16–18 for ROC plots, and Supplementary Figs. 19–21 for corresponding summary statistics).

### Evaluating balancing selection inference power under human demographic models

Balancing selection has arguably been most extensively studied in humans, and as such there is interest in how much power there is to detect balancing selection under commonly used models of human demography. Simulations were run under the Gravel *et al*. (2011) and Hu *et al*. (2023) models of human demography (see Supplementary Fig. 22 for schematics of demographic models). The Gravel *et al*. (2011) model is a 3-population model in which an African population increases in size, before the Eurasian population splits and experiences a population bottleneck. This is followed by the European and Asian populations splitting from one another and undergoing rapid expansion until the sampling time (Gravel *et al*. 2011). By contrast, the Hu *et al*. (2023) model is a single population model in which the African population undergoes a severe ancient bottleneck, before undergoing population expansion. Although the Out-of-Africa dispersal is modeled (Hu *et al*. 2023), it was not considered here, as balancing selection in the African population was of interest. See the *Materials and methods* section for details of how the two models were parameterized and simulated.

Principally an Out-of-Africa model, the Gravel model of human demography covers a relatively small space in evolutionary time (Gravel *et al*. 2011). After the 10*N* generation “burn-in” phase, the entire model is less than 1*N* generations in length. Simulations were therefore run with the balanced mutation introduced at different time points after the burn-in (with ∼0.01*N* generations between each time point—see Supplementary Figs. 23–25 for ROC plots, and Supplementary Fig. 26 for summary statistics). As expected, there was little power with the *B*_2_ method to infer balancing selection over these timescales, regardless of the population into which the balanced mutation was introduced, and which population was sampled. As already shown, SFS-based methods such as *B*_2_ have little power over such short timescales. A notable exception was that when the balanced mutation was introduced into the African population at ∼0.11*N* generations prior to sampling, this time point was reasonably well powered in both the European and East Asian populations. This time point is notable as it is immediately after the Eurasian population splits into the European and East Asian populations, both of which then undergo rapid exponential growth (Gravel *et al*. 2011). The *iHS* statistic performed as expected, being strongly powered when the balanced mutation was introduced near the sampling time, and unaffected by the severe demographic changes undergone by the European and East Asian populations—again due to the strong selection acting on the balance allele and conditioning on escaping stochastic loss. Simulations were additionally performed in the absence of balancing selection in order to quantify baseline false-positive rates (Supplementary Fig. 27). Both methods were largely robust to the human demographic models.

As the Hu *et al*. (2023) model of human demography reaches further back in time than the Gravel *et al*. (2011) model, covering over 10*N* generations with an ancient, severe bottleneck occurring in the African population, the balanced mutation was introduced at different times to evaluate inference power at different equilibrium frequencies of the balanced mutation (*f*_eq_ = 0.1, 0.25, 0.5, and 0.75). *B*_2_ was observed to have little power, even over these longer timescales (Supplementary Fig. 28; as shown above, until *τ_b_* ≥ 25*N* generations, SFS-based methods generally have little power to detect balancing selection, whilst *iHS* has very little power except on the shortest of timescales). As expected, power appears to be greatest at *f*_eq_ = 0.5 for *B*_2_, in which the balanced haplotype is segregating at an intermediate frequency. Deviation from this equilibrium frequency resulted in loss of power as the SFS skewed more toward higher or lower frequencies, as measured by the increased Tajima's *D* around the balanced mutation (Supplementary Fig. 29).

### Can population structure generate a signal of balancing selection?

It is well documented that population structure can confound balancing selection inference (Lewontin and Krakauer 1973; Charlesworth *et al*. 2003; Ingvarsson 2004; de Filippo *et al*. 2016; and see review of Bitarello *et al*. 2023). Coalescent simulations were run to evaluate the effects of population structure on balancing selection inference under three scenarios: (1) gene flow in which an ancestral population splits into two equally sized daughter populations, with 4*Nm* = 0, 0.01, 0.05, 0.1, or 0.2, before one population is sampled; (2) simulations in which an ancestral population splits into two equally sized daughter populations which undergo strong migration (4*Nm* = 0.25) for 5*N* generations, and are then isolated for the remaining 5*N* generations before sampling a single population; and (3) hidden structure simulations in which an ancestral population splits into two daughter populations which remain isolated for 5*N* generations, before both populations are equally sampled from (i.e. replicating a scenario of sampling across unknown population structure). For further details of these simulations see the *Materials and methods* section.

**Fig. 5.**
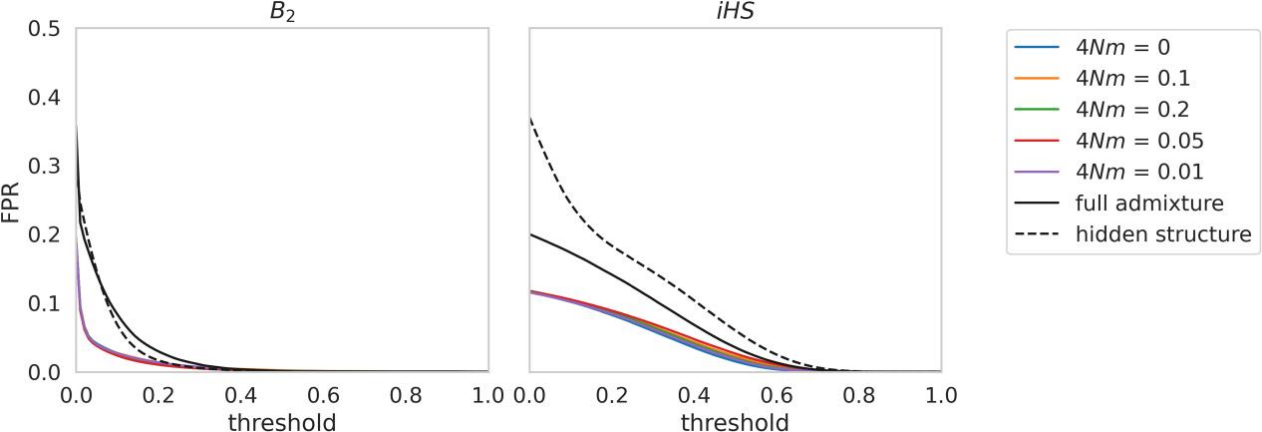
False-positive rates (FPRs) plotted across *B*_2_ and *iHS* thresholds under multiple population structure models, with and without gene flow or admixture. For gene flow simulations (those denoted by their 4*Nm* value in the figure legend), 2 populations were simulated with symmetric migration rates between them, where 4*Nm* is the population-scaled migration rate. For the full admixture simulations, an ancestral population was simulated, which split into 2 daughter populations, followed by strong gene flow (4*Nm* = 0.25). For the hidden structure simulations, an ancestral population was simulated, which split into 2 daughter populations that remained isolated for 5*N* generations, before sampling equally from each population. Because thresholds will depend on the minimum and maximum CLR or *iHS* values, these were normalized to between 0 and 1.

Gene flow alone was not observed to generate an excess of false positives, regardless of the migration rate (Fig. 5). However, both the admixture model and the hidden structure model generated considerable false positives with both *B*_2_ and *iHS*, emphasizing the confounding effects of these neutral processes. Populations that diverge before again merging can generate intermediate frequency haplotype blocks, resulting in an increase in false positives when inferring balancing selection. Similarly unaccounted for “hidden” structure can also generate linkage blocks at intermediate frequencies. Whilst the confounding effects of hidden structure have been acknowledged in terms of a single species occupying a large range in which different populations are being sampled and treated as a single population (Bitarello *et al*. 2023), results in this study provide a quantification of this impact. This result demonstrates the importance of first assessing population structure prior to performing population-specific demographic or selection analyses [e.g. using STRUCTURE (Hubisz *et al*. 2009) or ADMIXTURE (Alexander *et al*. 2009)].

### Concluding thoughts

Our results suggest that current approaches for detecting balancing selection in a single population are largely limited to relatively ancient balancing selection (>25*N* generations). Although LD-based approaches such as *iHS* are capable of detecting extremely recent balancing selection (≤0.1*N* generations), it is not possible to distinguish between partial selective sweeps and balancing selection at this time frame (Charlesworth 2006). Furthermore, SFS-based approaches were observed to be characterized by low power for considerable spans of time after the introduction of the balanced allele; indeed, it was not until *τ_b_* = 50*N* generations that SFS-based approaches were found to achieve a reasonable true-positive rate at a standard false-positive rate (e.g. TPR = 0.695 at FPR = 0.05), results that are in agreement with Cheng and DeGiorgio (2020, 2022). Assuming a human generation time of 26.9 years (Wang *et al*. 2023) and *N_e_* = 10,000, this suggests that there is little power to detect balancing selection in humans from population genomic data when the balanced mutation has been segregating for less than 25 × 10,000 × 26.9 = 6.725 million years. This timescale thus sits near what Bitarello *et al*. (2023) classified as ultra-long-term (>*T*_div_ + 4*N_e_* generations) balancing selection, where *T*_div_ is the divergence time between humans and chimpanzees [i.e. ∼6 million years (Patterson *et al*. 2006)]. Notably, oft-cited examples of balancing selection, such as at the MHC (Klein *et al*. 1998), ABO blood group (Ségurel *et al*. 2012) loci, and HLA genes (Leffler *et al*. 2013; Teixeira *et al*. 2015) are trans-species polymorphisms (i.e. sufficiently ancient such that SFS-based methods would be expected to be well-powered), though other variants implicated in malarial resistance for example (e.g. G6PD, estimated to be 620 KYA; Verrelli *et al*. 2002) are much younger and would thus be expected to be difficult to detect from population genetic data alone (Hedrick 2011).

Two alternative approaches that are in their infancy may provide additional traction in improving model identifiability: inference on multiple time point datasets rather than the more common single time point as focused upon here (Fijarczyk and Babik 2015), and the use of ancestral recombination graphs (ARGs) to capture allele frequency trajectories (Kelleher *et al*. 2019; Speidel *et al*. 2019; Stern *et al*. 2019). In the case of the former, the growing library of ancient DNA data presents a potential avenue for multiple time point analysis. For the latter, ARGs have already been implemented into methods for sweep detection (Stern *et al*. 2019), though the effects of purifying and background selection on such approaches remains an open question.

This study also highlights the importance of modeling an appropriate evolutionary baseline model when studying the power to detect balancing selection in any given population of interest. Accounting for population size change and structure, the purifying and background selection effects in and around functional genomic elements, and mutation and recombination rate heterogeneity across the genome, will facilitate the more accurate modeling of expected balancing selection patterns as well as expected true- and false-positive rates. Though a number of studies have investigated the effects of these processes in isolation (see DeGiorgio *et al*. 2014; Siewert and Voight 2017, 2020; Bitarello *et al*. 2018; Cheng and DeGiorgio 2020, 2022 for population structure and migration; and Bitarello *et al*. 2018; Cheng and DeGiorgio 2020, 2022; Isildak *et al*. 2021 for variable mutation and recombination rates), it is important to consider the joint and simultaneous contributions of these certain-to-be-operating evolutionary processes, as we have implemented here. The type of ultra-long-term balancing selection (as defined by Bitarello *et al*. 2023) for which sufficient power was observed in our study may well be rare relative to recent or transient balancing selection (<4*N_e_* generations). For example, some have made the case that short-lived heterozygote advantage may be relatively common as a pathway for novel adaptive variants (Sellis *et al*. 2011). Furthermore, Hill–Robertson effects can greatly reduce the probability of balanced alleles escaping stochastic loss, and the type of strong, long-term selective pressure necessary to maintain a balanced frequency over long evolutionary timescales may well be the exception rather than the norm. If this is indeed the case, our results suggest that current approaches are severely underpowered to detect more common instances of balancing selection. Though numerous methods exist for performing balancing selection inference (see reviews of Fijarczyk and Babik 2015; Bitarello *et al*. 2023), and are explicitly designed to infer either long-term or recent balancing selection, they generally rely fundamentally upon the SFS or LD-based patterns evaluated here, and will therefore likely be subject to the constraints described in this study.

A number of caveats are worth mentioning. Given that the strength of selection acting upon the balanced mutation was logically modeled as depending on its frequency in the population, the initial partial sweep phase of the trajectory was characterized by extremely strong selection, which naturally will only improve identifiability. Secondly, we generally assumed an equilibrium balanced mutation frequency of *f*_eq_ = 0.5. However, we observed that inference power was affected when this assumption was relaxed (Supplementary Figs. 28 and 29), and that the effect on inference power was dependent on the extent to which the equilibrium frequency deviated from 0.5. Thus, as with the above, this assumption likely only serves to maximize statistical power. Furthermore, we maintained the common assumption of *h* = 0.5, though the selective sweep dynamics of recessive and dominant mutations, and the impact on identifiability, have been well explored (Teshima *et al*. 2006). Finally, negative frequency-dependent selection was chosen in this study as an illustrative example, though one might also have chosen to model heterozygote advantage as others have done (e.g. Cheng and DeGiorgio 2020), or a model in which two balanced alleles interact in an additive manner (e.g. Glémin 2021). Here the focus was centered upon inference conditional on the balanced mutation establishing and reaching equilibrium frequency, with the stochastic loss simulations presented to emphasize that such establishment is naturally a low probability event.

Thus, at present, it remains difficult to clearly answer the central question of the *classical*/*balanced* debate: How much genetic variation is being maintained by balancing selection in natural populations? This study illustrates many of the challenges of balancing selection inference in humans, in many ways the most well-characterized study system in this debate. Specifically, by modeling an evolutionary baseline model consisting of a realistic distribution of fitness effects, population history, and underlying mutation and recombination rate variation—as has been recommended when performing such genomic scans (Johri, Eyre-Walker, *et al*. 2022; Johri, Aquadro, *et al*. 2022)—we primarily found power to detect relatively ancient, stably, and strongly balanced variants. Crucially however, even though we may not have the desirable level of resolution to address this important evolutionary question, this type of modeling and performance analysis at least quantifies identifiability and enables a reasonable description of expected false-positive rates, thereby reducing the risk of unfounded claims.

## Supplementary Material

jkae069_Supplementary_Data

## Data Availability

All scripts to generate and analyze simulated data, as well as to perform balancing selection inference, are available at the GitHub repository: https://github.com/vivaksoni/balancing_selection-temporal_challenges. Supplemental material available at G3 online.
