# Supplementary material for: Temporal challenges in detecting balancing selection from population genomic data
Source: G3 (Bethesda). 2024 Mar 29;14(6):jkae069. doi: 10.1093/g3journal/jkae069 (PMC11152078; doi:10.1093/g3journal/jkae069)
Supplement: jkae069_Supplementary_Data [file jkae069_supplementary_data.pdf]

S1

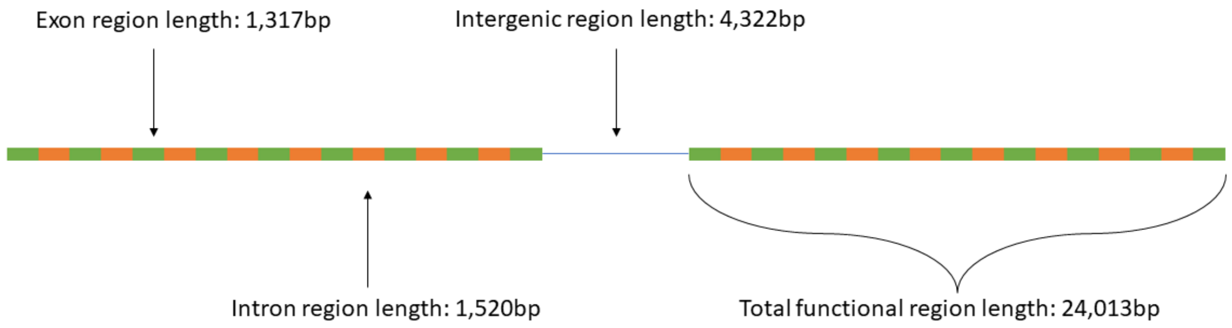

S1: Schematic of genic structure for DFE simulations. Each simulated region is made up of 3 functional regions, with each separated by an intergenic region.

S2

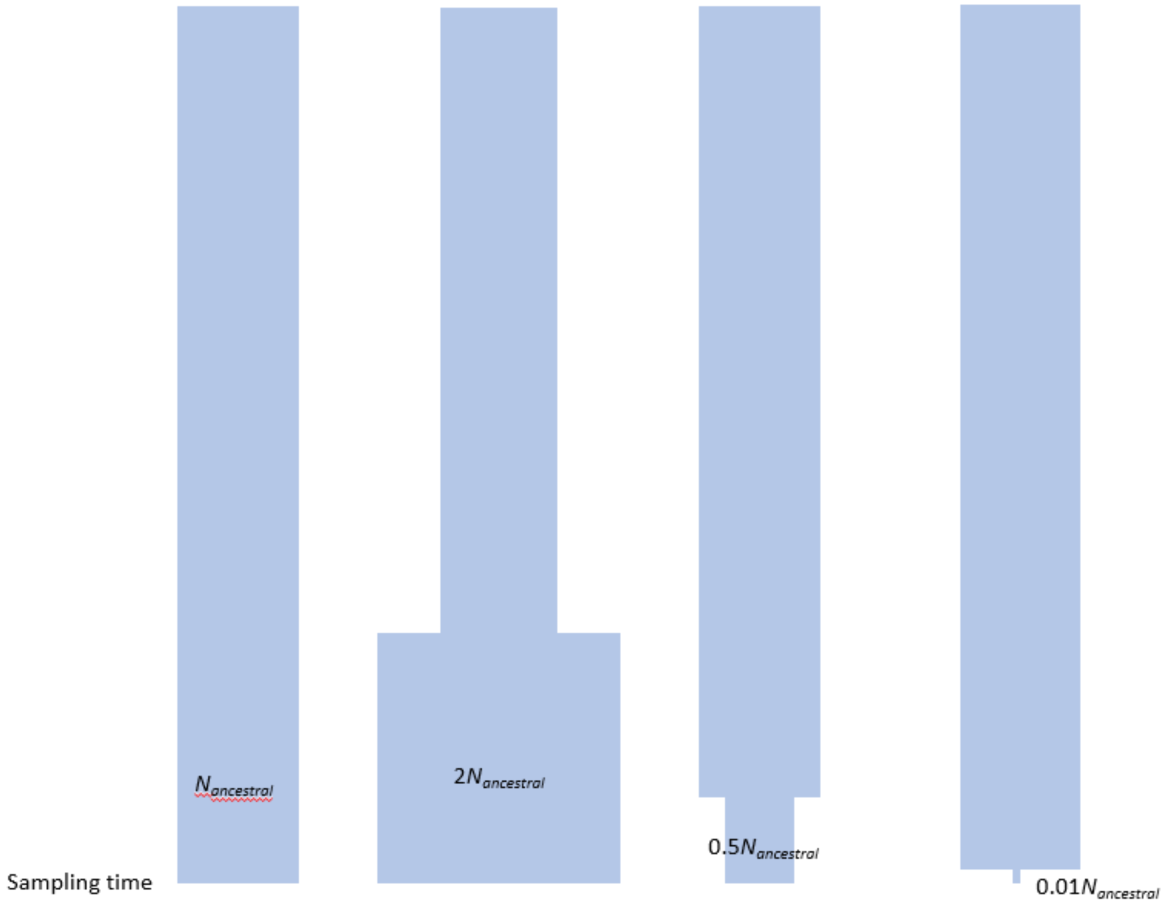

S2: 4 simulated demographic histories. In all scenarios a $10N_{ancestral}$ generation burn-in was simulated, where $N_{ancestral}$ is the initial population size. Population size change occurred instantaneously, $N_{current}$ generations before sampling, where $N_{current}$ is the population size following the size change.

S3

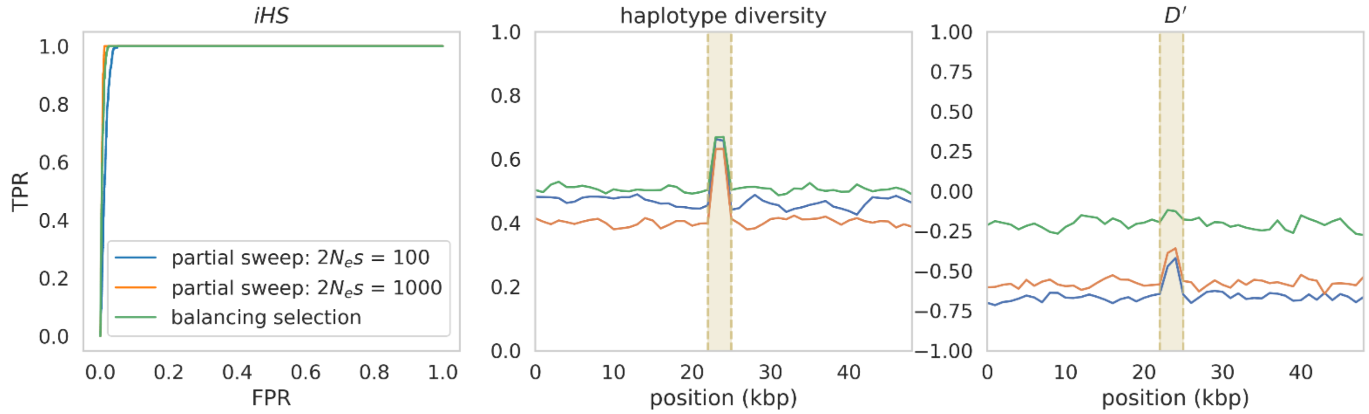

S3: *iHS* ROC curves (left) and summary statistics (haplotype diversity (middle), and mean *D'* (right)) for partial sweeps and balancing selection simulations of an equilibrium population on a neutral genetic background with fixed mutation and recombination rates. Balancing selection inference was performed at $\tau_b = 0.01N$, where τ_b is the time since introduction of the balanced mutation. For partial sweep simulations, a single beneficial mutation was introduced into the population after the 10*N* burn-in period. When the beneficial mutation reached a frequency of 0.5, the population was sampled. *iHS* inference was performed at each SNP, and ROC curves were generated using 100bp windows. For summary statistics, the shaded region represents windows in which the beneficial/balanced mutation is segregating. Summary statistics were estimated using a window size of 2kb and a step size of 1kb.

S4

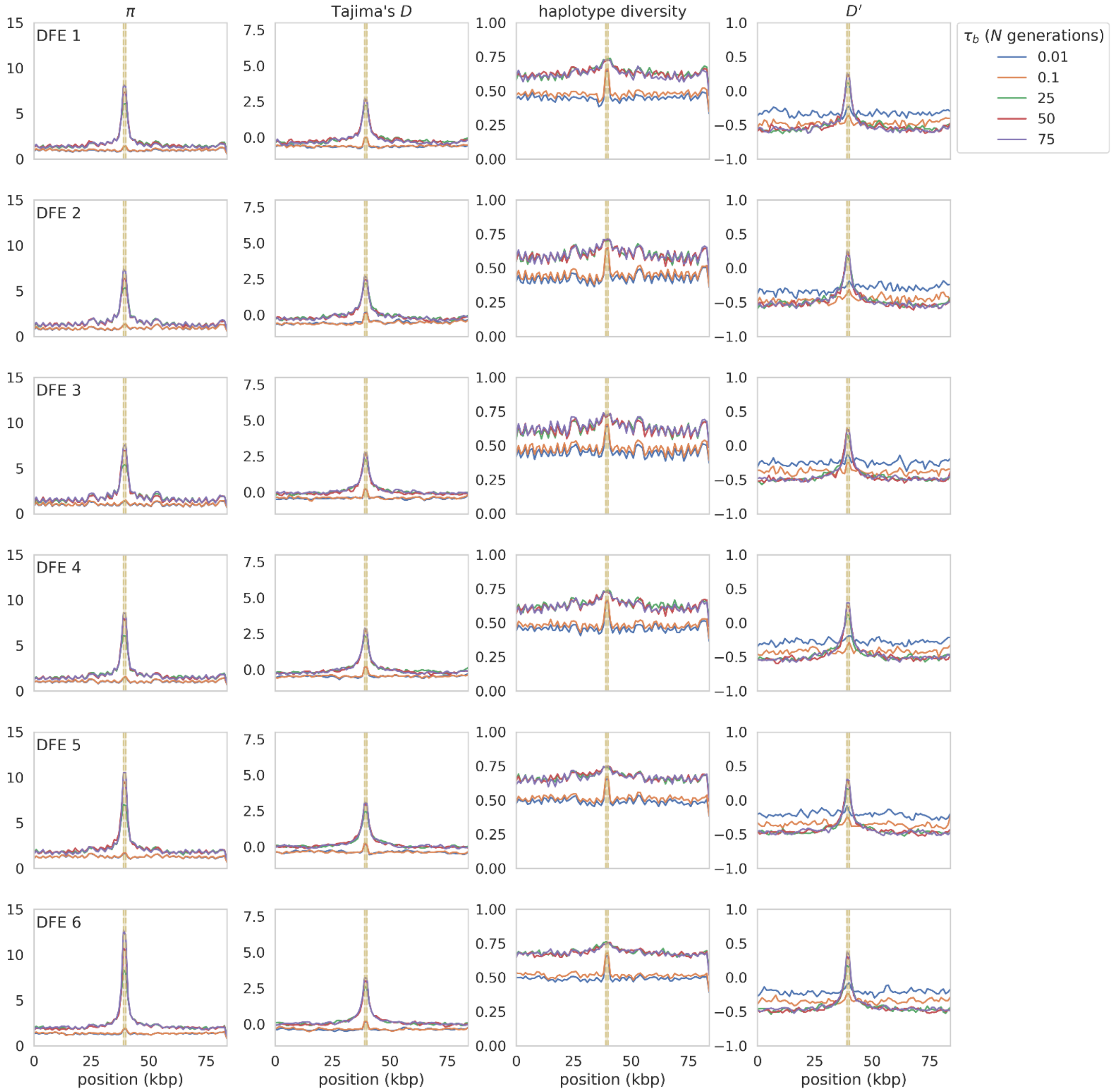

S4: Summary statistics for **equilibrium population** simulations with **fixed mutation and recombination rates** under 6 DFEs. The simulated population was sampled at a number of values of τ_b (time since the introduction of the balanced mutation). The shaded region represents windows in which the balanced mutation is segregating. Summary statistics were estimated using a window size of 2kb and a step size of 1kb. **These summary statistics correspond to inference results in Figure 2 of the main text**.

S5

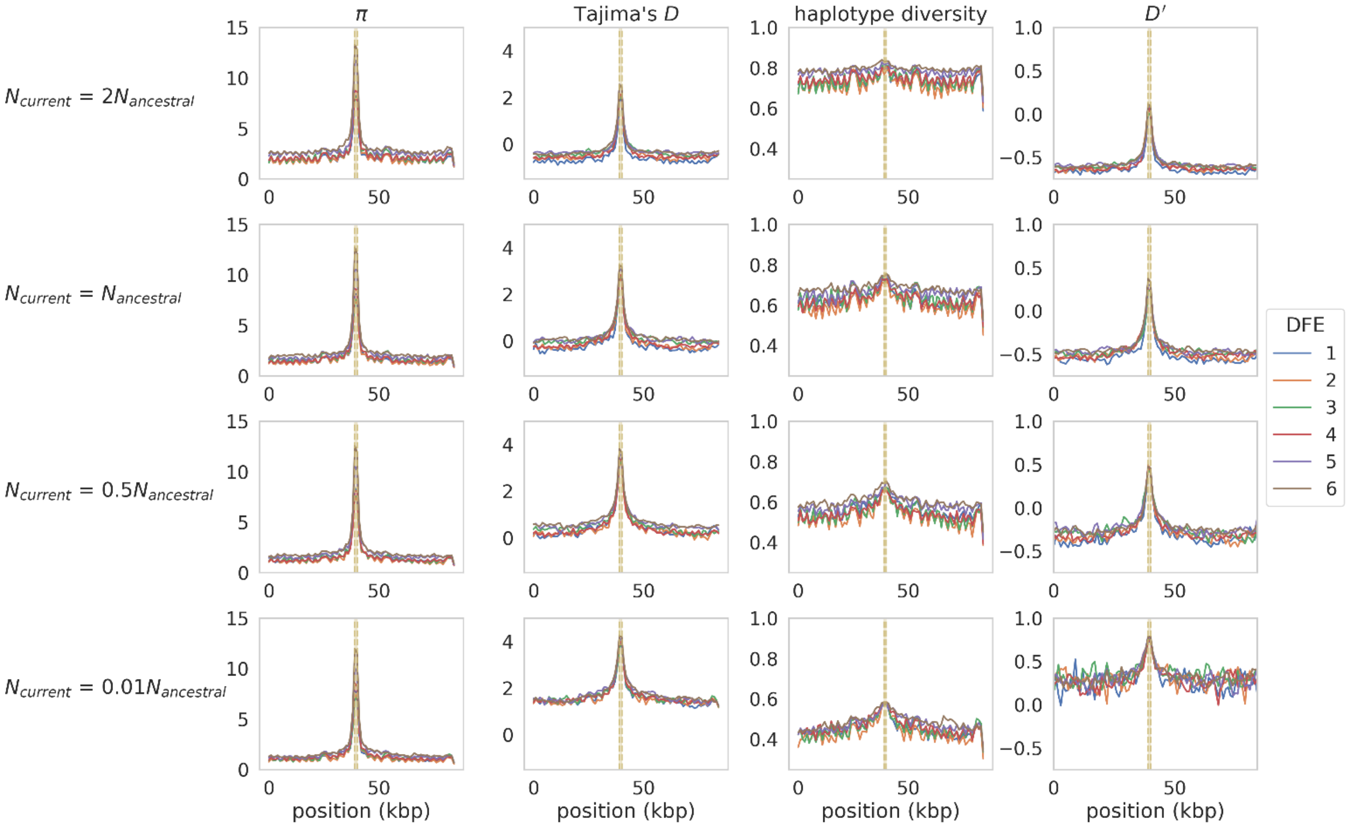

S5: Summary statistics for balancing selection simulations under 4 demographic histories across 6 DFEs, with fixed mutation and recombination rates across 200 simulated replicates. Population size change occurs $0.01N_{ancestral}$ generations before sampling (the same time as the introduction of the balanced mutation), where $N_{ancestral}$ was the initial population size. Exonic mutations were drawn from a DFE comprised of four fixed classes (following Johri et al. 2020), whose frequencies were denoted by f_i: f_0 with $0 \leq 2N_{ancestral}\, s < 1$ (*i.e.*, effectively neutral mutations), f_1 with $1 \leq 2N_{ancestral}\, s < 10$ (*i.e.*, weakly deleterious mutations), f_2 with $10 \leq 2N_{ancestral}\, s < 100$ (*i.e.*, moderately deleterious mutations), and f_3 with $100 \leq 2N_{ancestral}\, s$ (*i.e.*, strongly deleterious mutations), where s was the reduction in fitness of the mutant homozygote relative to wild-type. The shaded region represents windows in which the balanced mutation is segregating. Summary statistics were estimated using a window size of 2kb and a step size of 1kb. **These summary statistics correspond to inference results in the top row of Figure 3 of the main text**.

S6

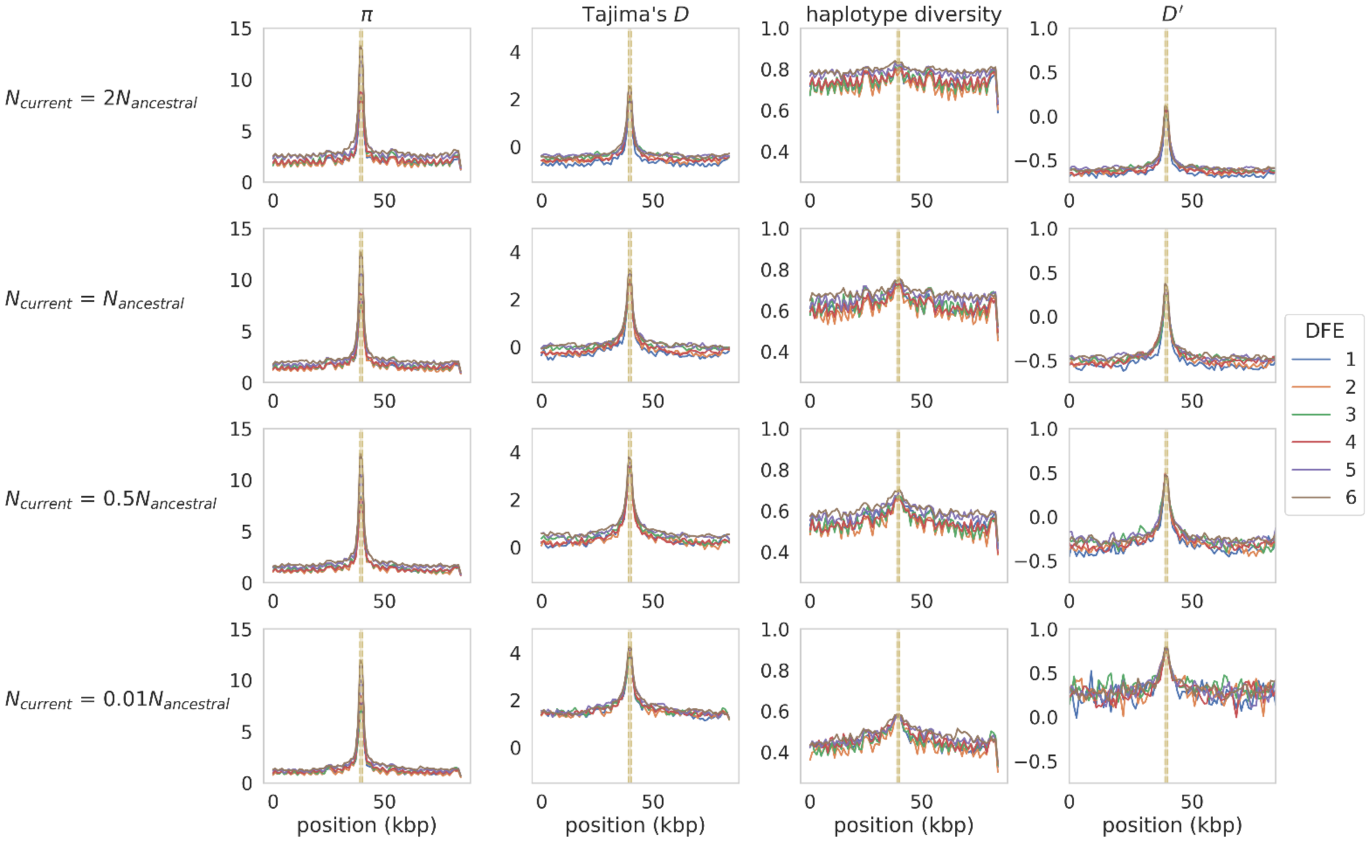

S6: Summary statistics for balancing selection simulations under 4 demographic histories across 6 DFEs, with fixed mutation and recombination rates across 200 simulated replicates. Population size change occurred $N_{current}$ generations before sampling, where $N_{current}$ was the population size at time of sampling. Sampling occurred $75N_{ancestral}$ generations after the introduction of the balanced mutation, where $N_{ancestral}$ was the initial population size. Exonic mutations were drawn from a DFE comprised of four fixed classes (following Johri et al. 2020), whose frequencies were denoted by f_i: f_0 with $0 \leq 2N_{ancestral}\, s < 1$ (*i.e.*, effectively neutral mutations), f_1 with $1 \leq 2N_{ancestral}\, s < 10$ (*i.e.*, weakly deleterious mutations), f_2 with $10 \leq 2N_{ancestral}\, s < 100$ (*i.e.*, moderately deleterious mutations), and f_3 with $100 \leq 2N_{ancestral}\, s$ (*i.e.*, strongly deleterious mutations), where s was the reduction in fitness of the mutant homozygote relative to wild-type. The shaded region represents windows in which the balanced mutation is segregating. Summary statistics were estimated using a window size of 2kb and a step size of 1kb. **These summary statistics correspond to inference results in the top row of Figure 3 of the main text**.

S7

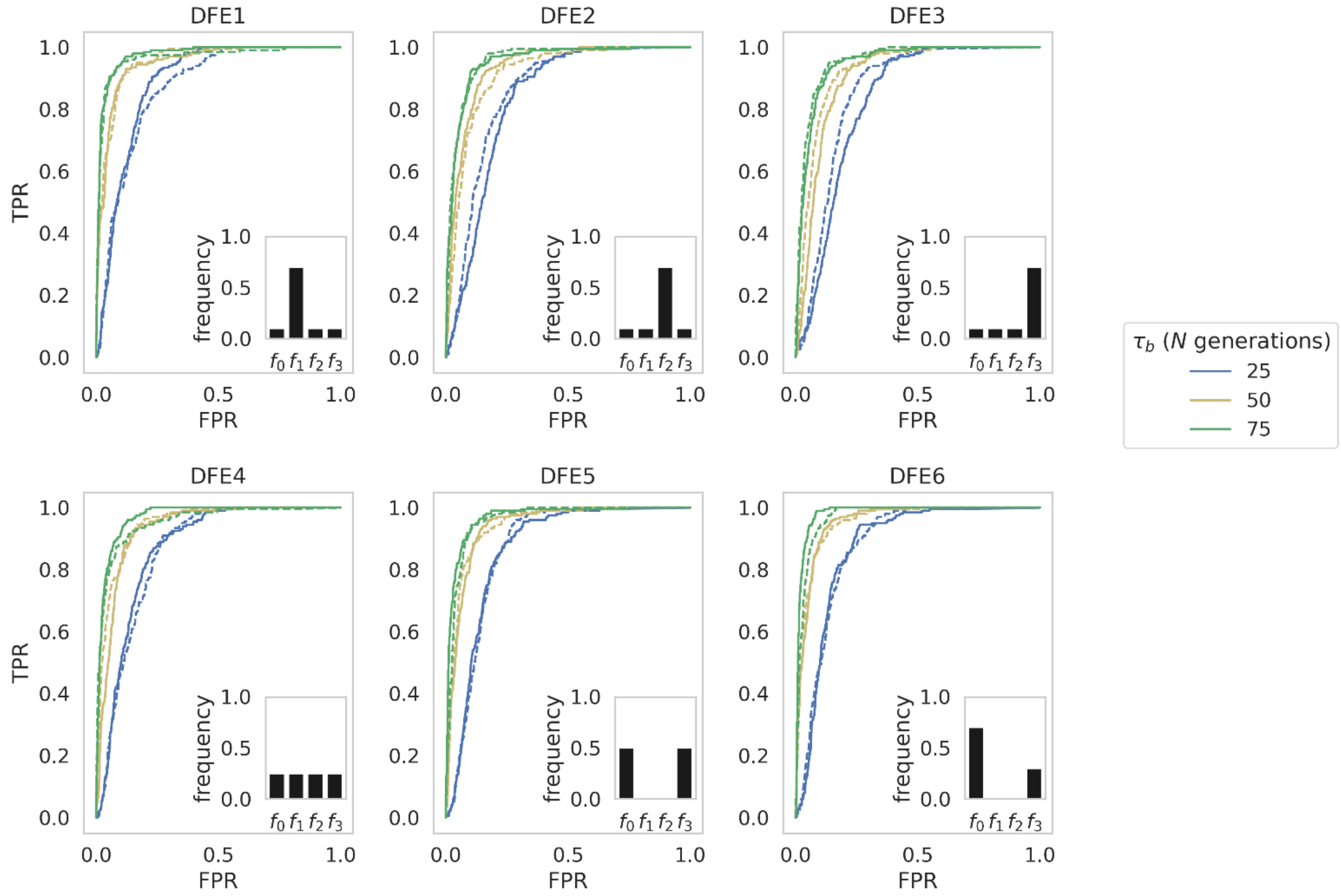

S7: ROC curves presenting the change in true-positive rate (TPR) as the false-positive rate (FPR) increases, for balancing selection inference when **recombination rates are fixed and mutation rates are variable** (dashed lines), compared with fixed rates (solid lines). Simulations were performed for an equilibrium population, under 6 DFEs, across 200 simulated replicates, using the $\boldsymbol{B_2}$ **method**. Timescales for inference were limited to those in which the method performed best: 25*N,* 50*N,* and 75*N* generations. B_2 inference was performed at each SNP, and ROC curves were generated using 100bp windows. Inlayed plots show the discrete DFE used for each panel. Exonic mutations were drawn from a DFE comprised of four fixed classes (following Johri et al. 2020), whose frequencies were denoted by f_i: f_0 with $0 \leq 2N_{ancestral}\ s < 1$ (*i.e.*, effectively neutral mutations), f_1 with $1 \leq 2N_{ancestral}\ s < 10$ (*i.e.*, weakly deleterious mutations), f_2 with $10 \leq 2N_{ancestral}\ s < 100$ (*i.e.*, moderately deleterious mutations), and f_3 with $100 \leq 2N_{ancestral}\ s$ (*i.e.*, strongly deleterious mutations), where *s* was the reduction in fitness of the mutant homozygote relative to wild-type. For variable rates, each 1kb region has a rate drawn from a uniform distribution such that each simulated replicate has the same mean rate as the fixed rate comparison (see Methods section for further details).

S8

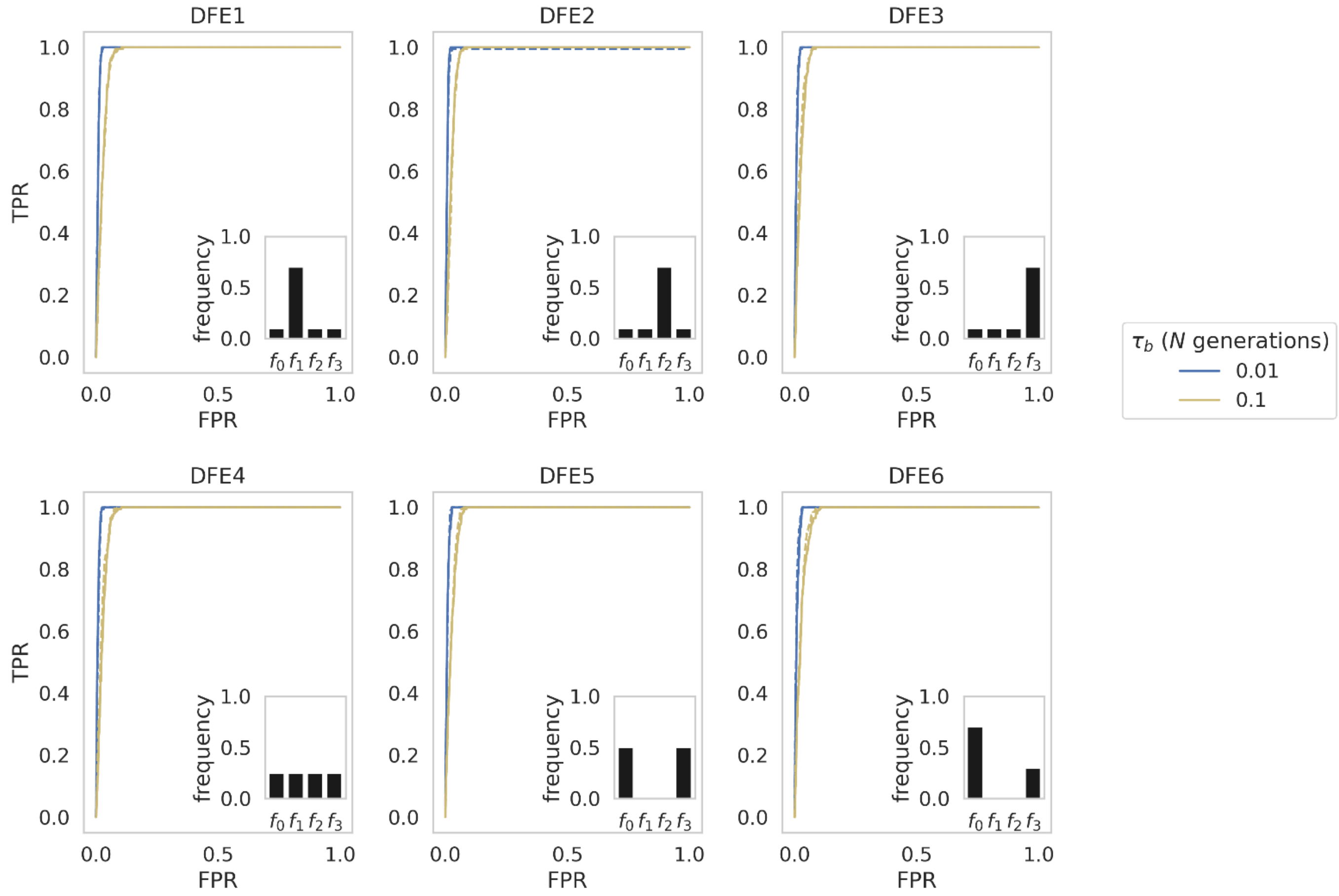

S8: ROC curves showing the change in true-positive rate (TPR) as the false-positive rate (FPR) increases, comparing balancing selection inference when **recombination rates are fixed and mutation rates are variable** (dashed lines), compared with fixed rates (solid lines). Simulations were performed for an equilibrium population, under 6 DFEs, across 200 simulated replicates, using the ***iHS* method**. Timescales for inference were limited to those in which each method performed best: 0.01*N* and 0.1*N* generations for *iHS*. *iHS* inference was performed at each SNP, and ROC curves were generated using 100bp windows. Inlayed plots show the discrete DFE for each panel. Exonic mutations were drawn from a DFE comprised of four fixed classes (following Johri et al. 2020), whose frequencies were denoted by f_i: f_0 with $0 \leq 2N_{ancestral}\, s < 1$ (*i.e.*, effectively neutral mutations), f_1 with $1 \leq 2N_{ancestral}\, s < 10$ (*i.e.*, weakly deleterious mutations), f_2 with $10 \leq 2N_{ancestral}\, s < 100$ (*i.e.*, moderately deleterious mutations), and f_3 with $100 \leq 2N_{ancestral}\, s$ (*i.e.*, strongly deleterious mutations), where *s* was the reduction in fitness of the mutant homozygote relative to wild-type. For variable rates, each 1kb region has a rate drawn from a uniform distribution such that each simulated replicate has the same mean rate as the fixed rate comparison (see Methods section for further details).

S9

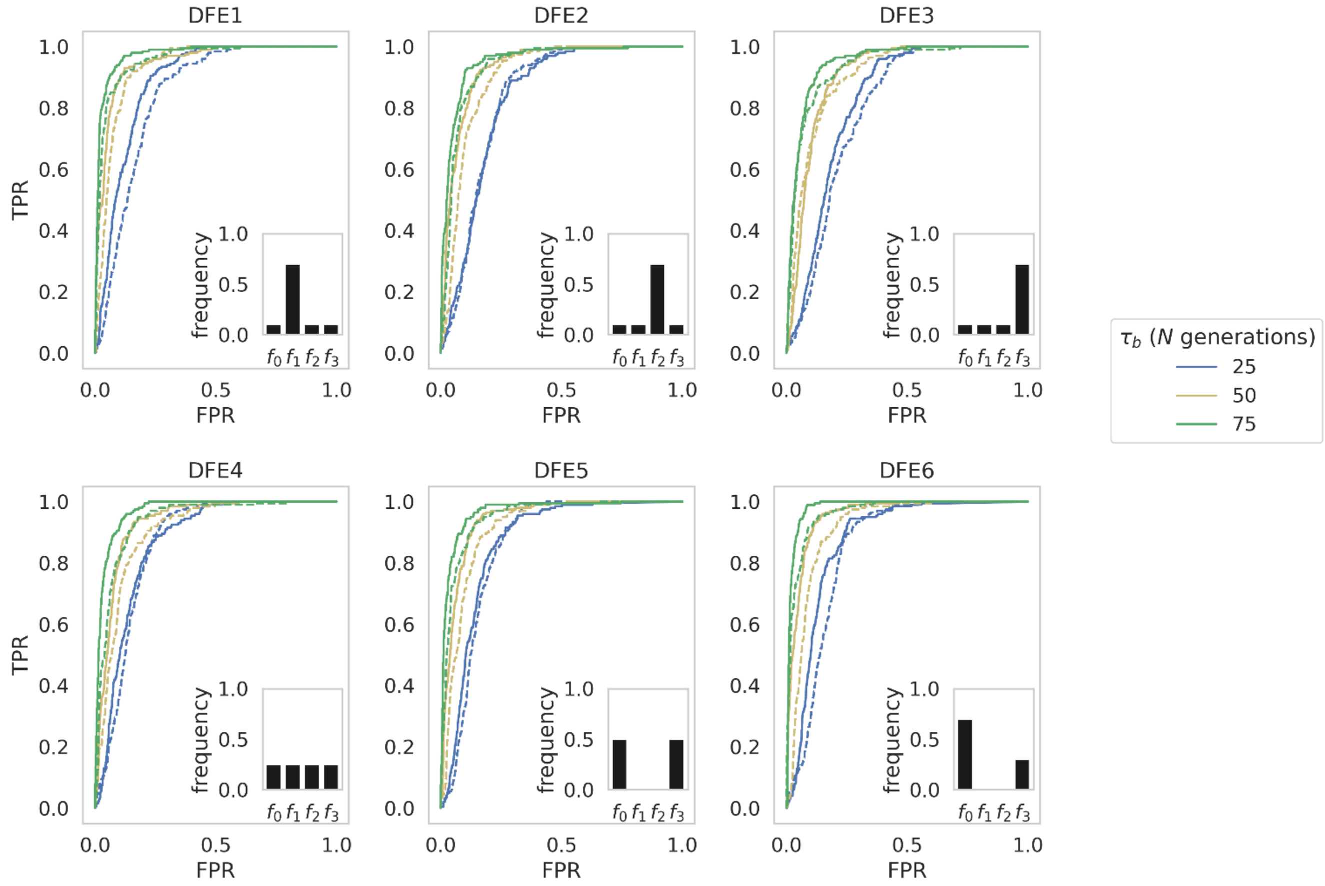

S9: ROC curves presenting the change in true-positive rate (TPR) as the false-positive rate (FPR) increases, for balancing selection inference when **recombination rates are variable and mutation rates are fixed** (dashed lines), compared with fixed rates (solid lines). Simulations were performed for an equilibrium population, under 6 DFEs, across 200 simulated replicates, using the $\boldsymbol{B_2}$ **method**. Timescales for inference were limited to those in which the method performed best: 25*N,* 50*N,* and 75*N* generations. B_2 inference was performed at each SNP, and ROC curves were generated using 100bp windows. Inlayed plots show the discrete DFE used for each panel. Exonic mutations were drawn from a DFE comprised of four fixed classes (following Johri et al. 2020), whose frequencies were denoted by f_i: f_0 with $0 \leq 2N_{ancestral}\, s < 1$ (*i.e.*, effectively neutral mutations), f_1 with $1 \leq 2N_{ancestral}\, s < 10$ (*i.e.*, weakly deleterious mutations), f_2 with $10 \leq 2N_{ancestral}\, s < 100$ (*i.e.*, moderately deleterious mutations), and f_3 with $100 \leq 2N_{ancestral}\, s$ (*i.e.*, strongly deleterious mutations), where *s* was the reduction in fitness of the mutant homozygote relative to wild-type. For variable rates, each 1kb region has a rate drawn from a uniform distribution such that each simulated replicate has the same mean rate as the fixed rate comparison (see Methods section for further details).

S10

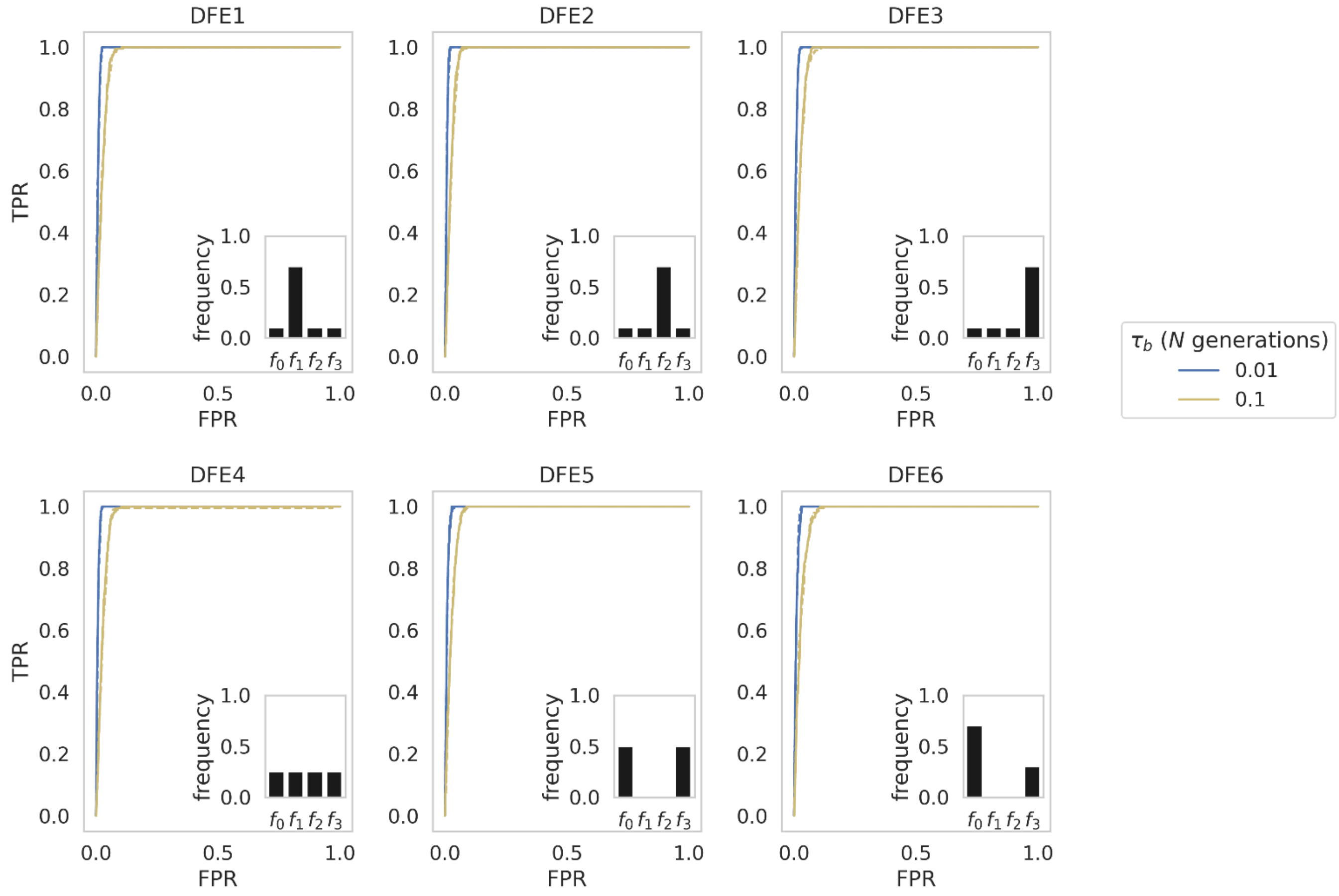

S10: ROC curves showing the change in true-positive rate (TPR) as the false-positive rate (FPR) increases, comparing balancing selection inference when **recombination rates are variable and mutation rates are fixed** (dashed lines), compared with fixed rates (solid lines). Simulations were performed for an equilibrium population, under 6 DFEs, across 200 simulated replicates, using the ***iHS* method**. Timescales for inference were limited to those in which each method performed best: 0.01*N* and 0.1*N* generations for *iHS*. *iHS* inference was performed at each SNP, and ROC curves were generated using 100bp windows. Inlayed plots show the discrete DFE for each panel. Exonic mutations were drawn from a DFE comprised of four fixed classes (following Johri et al. 2020), whose frequencies were denoted by f_i: f_0 with $0 \leq 2N_{ancestral}\, s < 1$ (*i.e.*, effectively neutral mutations), f_1 with $1 \leq 2N_{ancestral}\, s < 10$ (*i.e.*, weakly deleterious mutations), f_2 with $10 \leq 2N_{ancestral}\, s < 100$ (*i.e.*, moderately deleterious mutations), and f_3 with $100 \leq 2N_{ancestral}\, s$ (*i.e.*, strongly deleterious mutations), where *s* was the reduction in fitness of the mutant homozygote relative to wild-type. For variable rates, each 1kb region has a rate drawn from a uniform distribution such that each simulated replicate has the same mean rate as the fixed rate comparison (see Methods section for further details).

S11

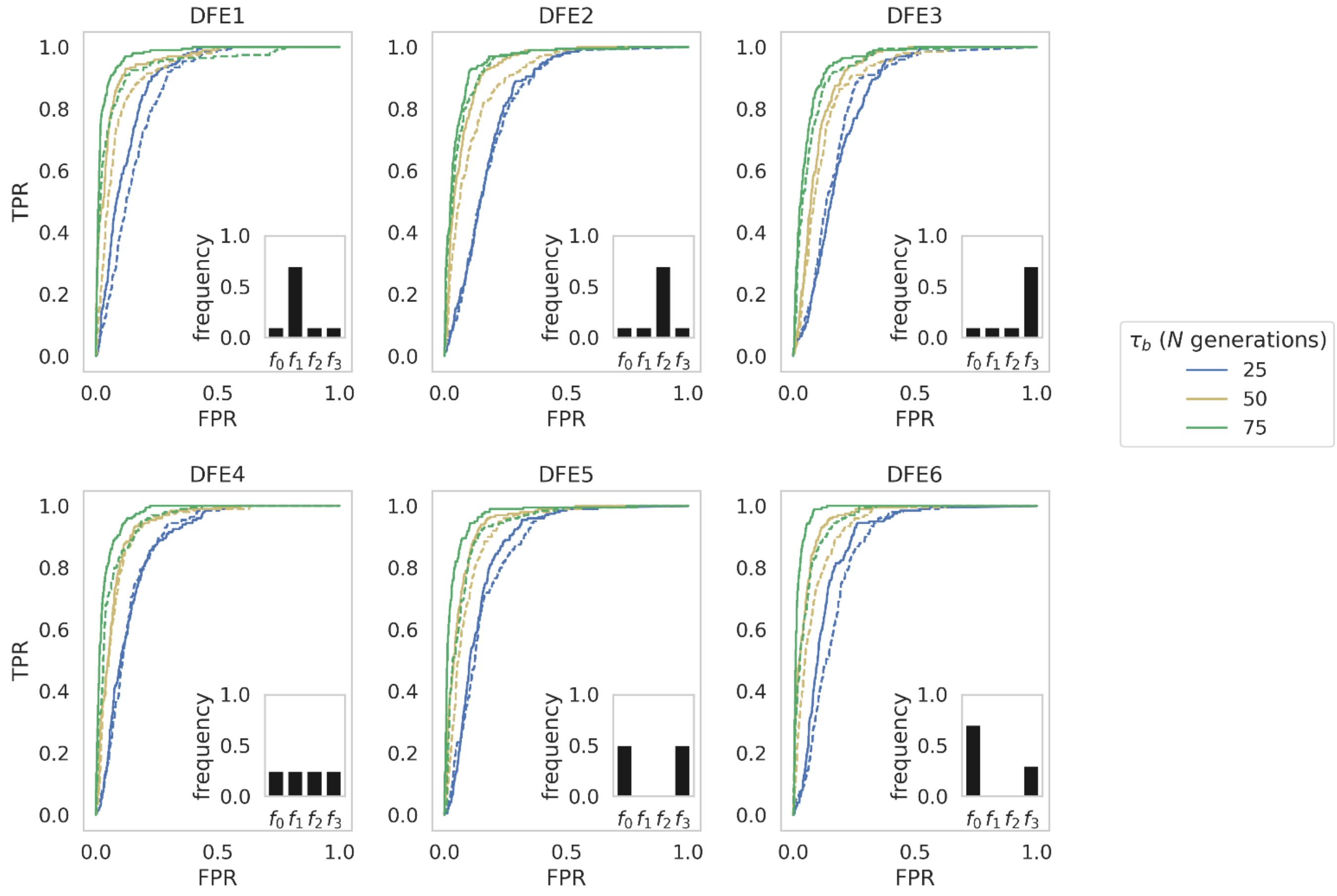

S11: ROC curves presenting the change in true-positive rate (TPR) as the false-positive rate (FPR) increases, for balancing selection inference when **recombination rates are variable and mutation rates are variable** (dashed lines), compared with fixed rates (solid lines). Simulations were performed for an equilibrium population, under 6 DFEs, across 200 simulated replicates, using the ***B_2* method**. Timescales for inference were limited to those in which the method performed best: 25*N,* 50*N,* and 75*N* generations. B_2 inference was performed at each SNP, and ROC curves were generated using 100bp windows. Inlayed plots show the discrete DFE used for each panel. Exonic mutations were drawn from a DFE comprised of four fixed classes (following Johri et al. 2020), whose frequencies were denoted by f_i: f_0 with $0 \le 2N_{ancestral}\, s < 1$ (*i.e.*, effectively neutral mutations), f_1 with $1 \le 2N_{ancestral}\, s < 10$ (*i.e.*, weakly deleterious mutations), f_2 with $10 \le 2N_{ancestral}\, s < 100$ (*i.e.*, moderately deleterious mutations), and f_3 with $100 \le 2N_{ancestral}\, s$ (*i.e.*, strongly deleterious mutations), where *s* was the reduction in fitness of the mutant homozygote relative to wild-type. For variable rates, each 1kb region has a rate drawn from a uniform distribution such that each simulated replicate has the same mean rate as the fixed rate comparison (see Methods section for further details).

S12

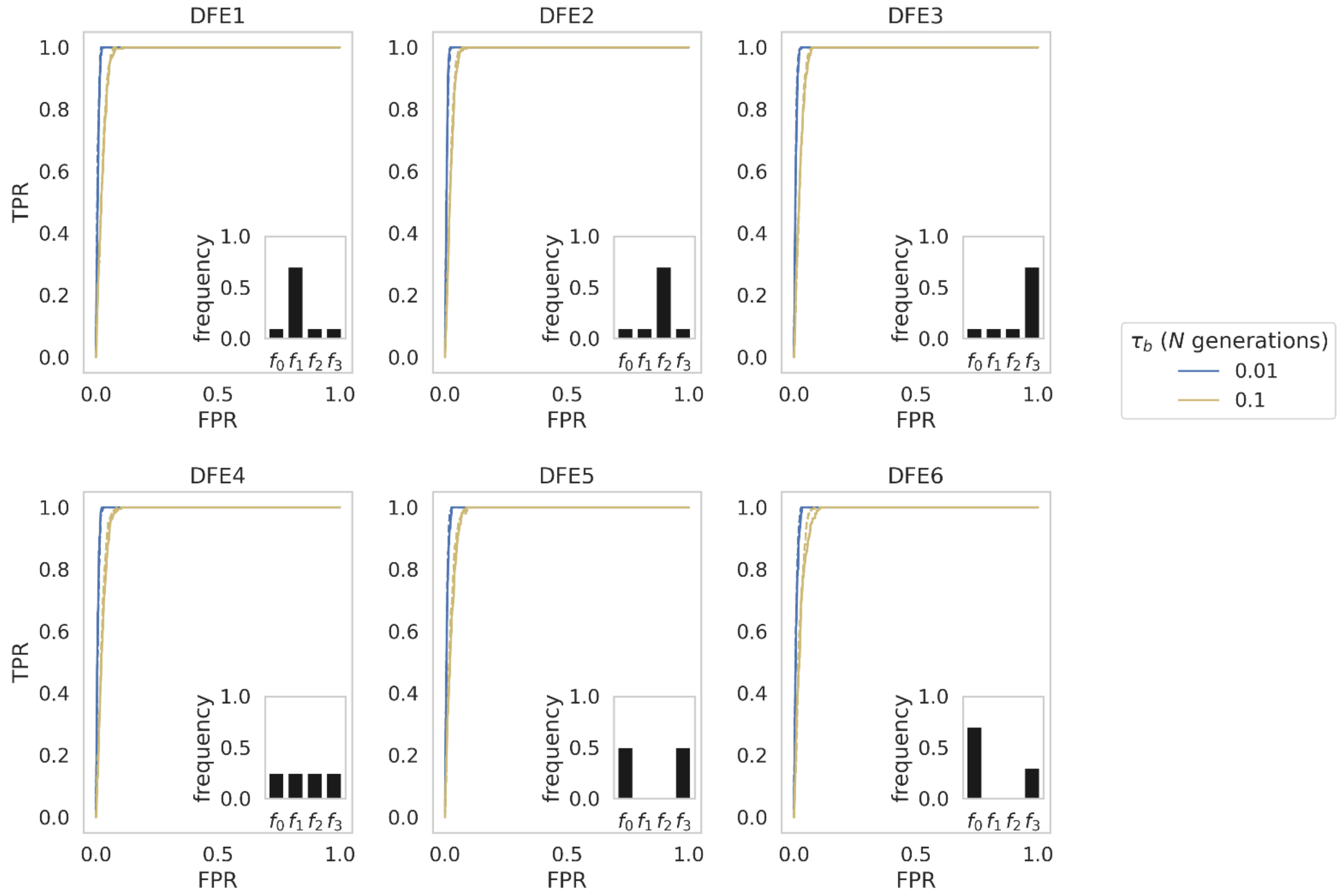

S12: ROC curves showing the change in true-positive rate (TPR) as the false-positive rate (FPR) increases, comparing balancing selection inference when **recombination rates are variable and mutation rates are variable** (dashed lines), compared with fixed rates (solid lines). Simulations were performed for an equilibrium population, under 6 DFEs, across 200 simulated replicates, using the ***iHS* method**. Timescales for inference were limited to those in which each method performed best: 0.01N and 0.1N generations for *iHS*. *iHS* inference was performed at each SNP, and ROC curves were generated using 100bp windows. Inlayed plots show the discrete DFE for each panel. Exonic mutations were drawn from a DFE comprised of four fixed classes (following Johri et al. 2020), whose frequencies were denoted by f_i: f_0 with $0 \leq 2N_{ancestral}\, s < 1$ (*i.e.*, effectively neutral mutations), f_1 with $1 \leq 2N_{ancestral}\, s < 10$ (*i.e.*, weakly deleterious mutations), f_2 with $10 \leq 2N_{ancestral}\, s < 100$ (*i.e.*, moderately deleterious mutations), and f_3 with $100 \leq 2N_{ancestral}\, s$ (*i.e.*, strongly deleterious mutations), where s was the reduction in fitness of the mutant homozygote relative to wild-type. For variable rates, each 1kb region has a rate drawn from a uniform distribution such that each simulated replicate has the same mean rate as the fixed rate comparison (see Methods section for further details).

S13

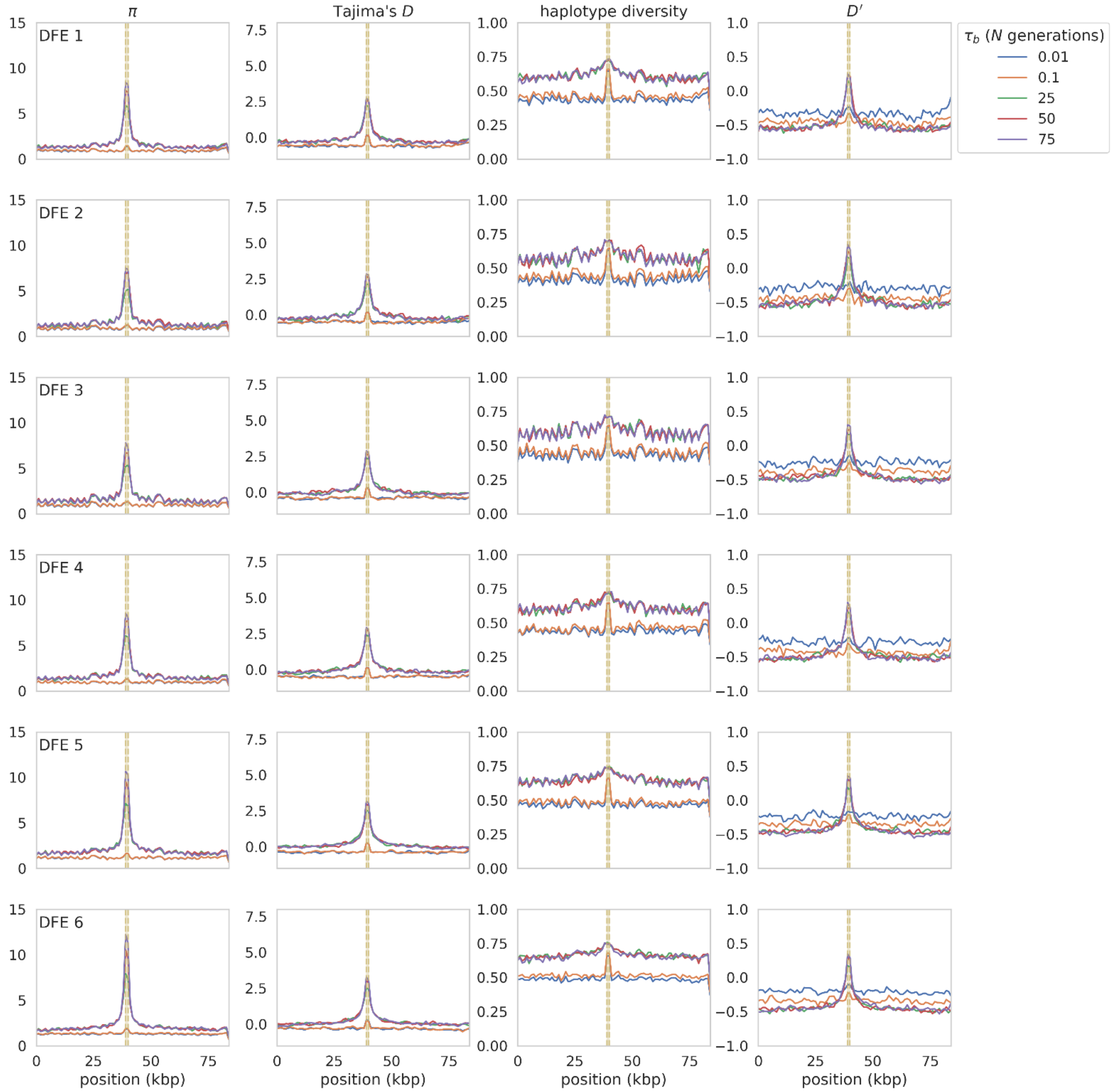

S13: Summary statistics for **equilibrium population** simulations with **variable mutation and fixed recombination rates** under 6 DFEs. The simulated population was sampled at a number of values of τ_b (time since the introduction of the balanced mutation). The shaded region represents windows in which the balanced mutation is segregating. Summary statistics were estimated using a window size of 2kb and a step size of 1kb. For variable rates, each 1kb region has a rate drawn from a uniform distribution such that each simulated replicate has the same mean rate as the fixed rate comparison (see Methods section for further details).

S14

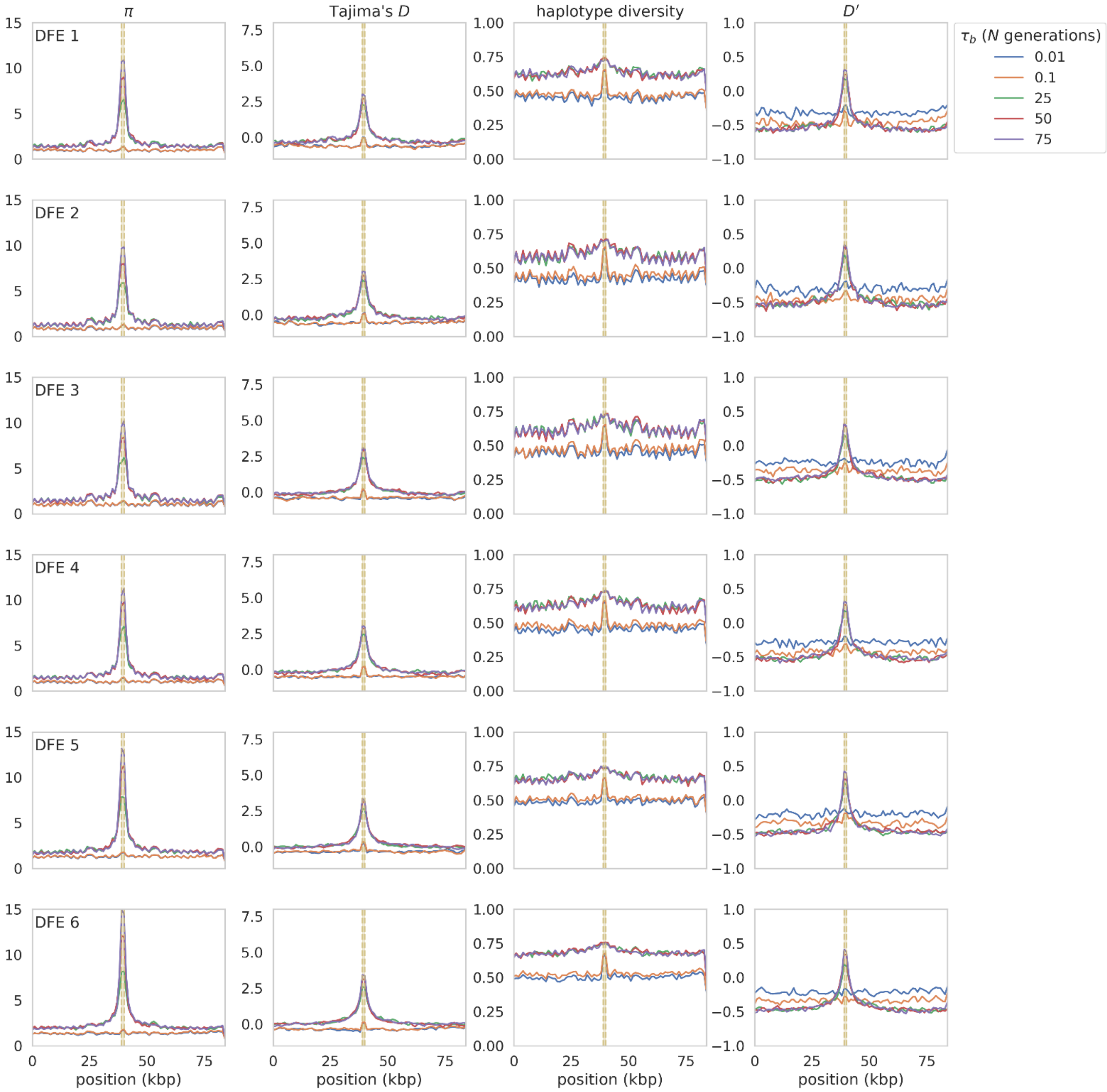

S14: Summary statistics for **equilibrium population** simulations with **fixed mutation and variable recombination rates** under 6 DFEs. The simulated population was sampled at a number of values of τ_b (time since the introduction of the balanced mutation). The shaded region represents windows in which the balanced mutation is segregating. Summary statistics were estimated using a window size of 2kb and a step size of 1kb. For variable rates, each 1kb region has a rate drawn from a uniform distribution such that each simulated replicate has the same mean rate as the fixed rate comparison (see Methods section for further details).

S15

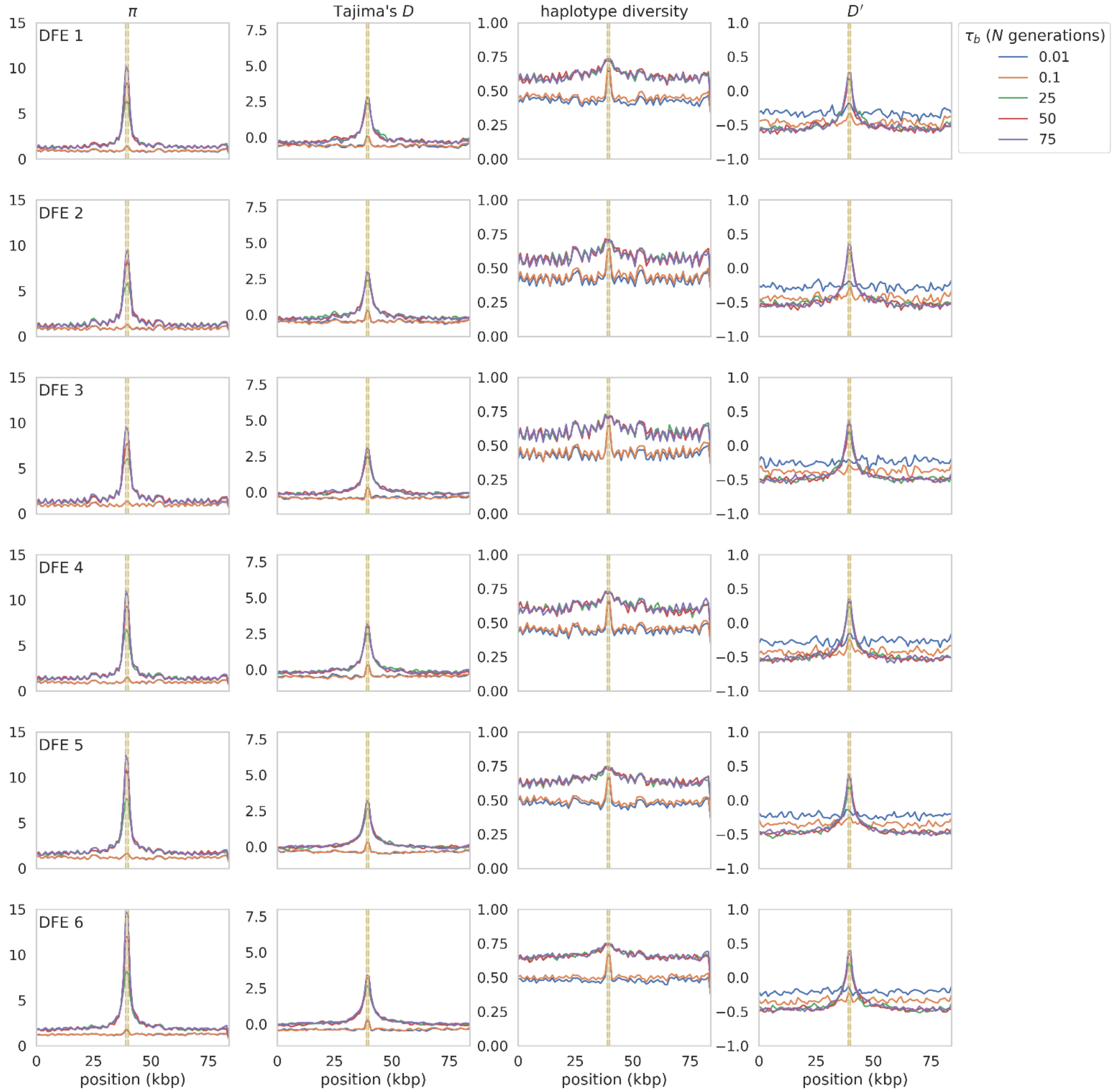

S15: Summary statistics for **equilibrium population** simulations with **variable mutation and variable recombination rates** under 6 DFEs. The simulated population was sampled at a number of values of τ_b (time since the introduction of the balanced mutation). The shaded region represents windows in which the balanced mutation is segregating. Summary statistics were estimated using a window size of 2kb and a step size of 1kb. For variable rates, each 1kb region has a rate drawn from a uniform distribution such that each simulated replicate has the same mean rate as the fixed rate comparison (see Methods section for further details).

S16

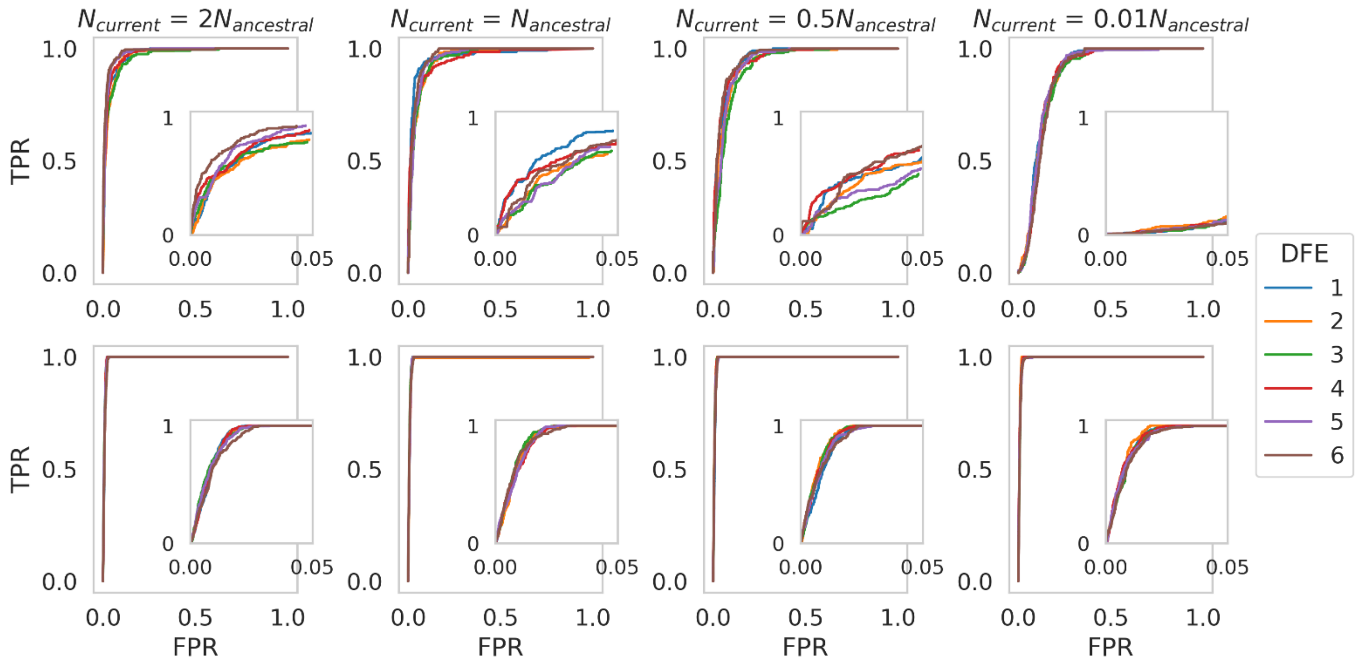

S16: ROC curves presenting the change in true-positive rate (TPR) as the false-positive rate (FPR) increases, for balancing selection inference under 4 demographic histories across 6 DFEs, with **fixed recombination and variable mutation rates** across 200 simulated replicates, using the B_2 method and the *iHS* statistic. In each case, population size change was instantaneous. For variable rates, each 1kb region has a rate drawn from a uniform distribution such that each simulated replicate has the same mean rate as the fixed rate comparison (see Methods section for further details). Top row: B_2 inference results for simulations in which population size change occurred $N_{current}$ generations before sampling, where $N_{current}$ was the population size at time of sampling. Sampling occurred $75N_{ancestral}$ generations after the introduction of the balanced mutation, where $N_{ancestral}$ was the initial population size. Bottom row: *iHS* inference results for simulations in which the population size change occurs $0.01N_{ancestral}$ generations before sampling (the same time as the introduction of the balanced mutation). Inset plots show zoomed in ROC curves covering FPR values from 0 to 0.05. *iHS* and B_2 inference were performed at each SNP, and ROC curves were generated using 100bp windows. Exonic mutations were drawn from a DFE comprised of four fixed classes (following Johri et al. 2020), whose frequencies were denoted by f_i: f_0 with $0 \leq 2N_{ancestral}\,s < 1$ (*i.e.*, effectively neutral mutations), f_1 with $1 \leq 2N_{ancestral}\,s < 10$ (*i.e.*, weakly deleterious mutations), f_2 with $10 \leq 2N_{ancestral}\,s < 100$ (*i.e.,* moderately deleterious mutations), and f_3 with $100 \leq 2N_{ancestral}\,s$ (*i.e.*, strongly deleterious mutations), where s was the reduction in fitness of the mutant homozygote relative to wild-type. See Figure 2 for plots of DFEs.

S17

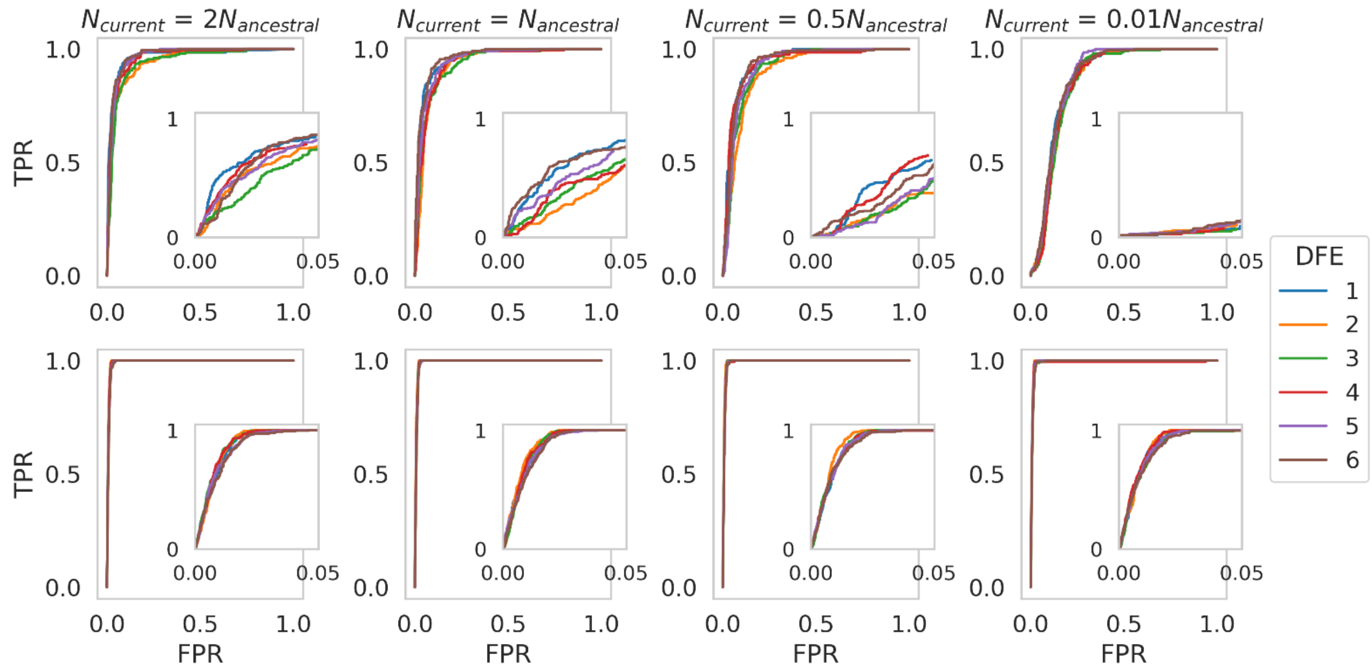

S17: ROC curves presenting the change in true-positive rate (TPR) as the false-positive rate (FPR) increases, for balancing selection inference under 4 demographic histories across 6 DFEs, with **variable recombination and fixed mutation rates** across 200 simulated replicates, using the B_2 method and the *iHS* statistic. In each case, population size change was instantaneous. For variable rates, each 1kb region has a rate drawn from a uniform distribution such that each simulated replicate has the same mean rate as the fixed rate comparison (see Methods section for further details). Top row: B_2 inference results for simulations in which population size change occurred $N_{current}$ generations before sampling, where $N_{current}$ was the population size at time of sampling. Sampling occurred $75N_{ancestral}$ generations after the introduction of the balanced mutation, where $N_{ancestral}$ was the initial population size. Bottom row: *iHS* inference results for simulations in which the population size change occurs $0.01N_{ancestral}$ generations before sampling (the same time as the introduction of the balanced mutation). Inset plots show zoomed in ROC curves covering FPR values from 0 to 0.05. *iHS* and B_2 inference were performed at each SNP, and ROC curves were generated using 100bp windows. Exonic mutations were drawn from a DFE comprised of four fixed classes (following Johri et al. 2020), whose frequencies were denoted by f_i: f_0 with $0 \leq 2N_{ancestral}\, s < 1$ (*i.e.*, effectively neutral mutations), f_1 with $1 \leq 2N_{ancestral}\, s < 10$ (*i.e.*, weakly deleterious mutations), f_2 with $10 \leq 2N_{ancestral}\, s < 100$ (*i.e.,* moderately deleterious mutations), and f_3 with $100 \leq 2N_{ancestral}\, s$ (*i.e.*, strongly deleterious mutations), where *s* was the reduction in fitness of the mutant homozygote relative to wild-type. See Figure 2 for plots of DFEs.

S18

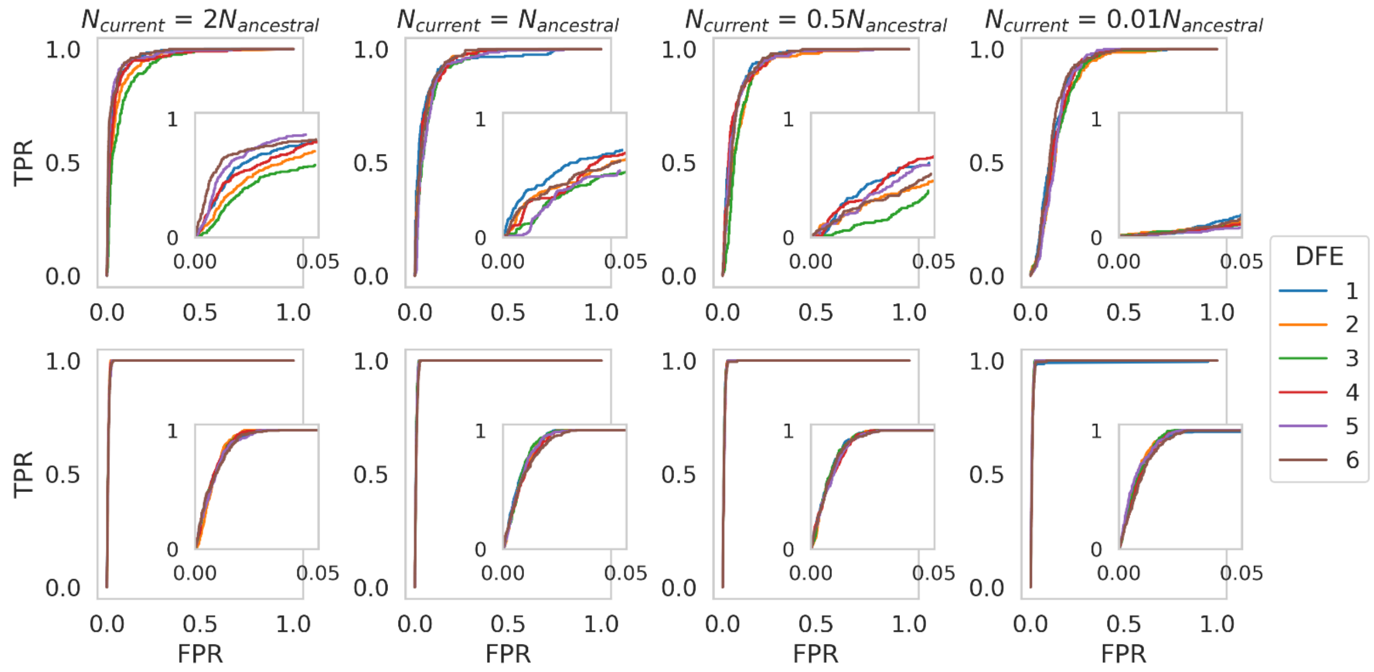

S18: ROC curves presenting the change in true-positive rate (TPR) as the false-positive rate (FPR) increases, for balancing selection inference under 4 demographic histories across 6 DFEs, with **variable recombination and variable mutation rates** across 200 simulated replicates, using the B_2 method and the *iHS* statistic. In each case, population size change was instantaneous. For variable rates, each 1kb region has a rate drawn from a uniform distribution such that each simulated replicate has the same mean rate as the fixed rate comparison (see Methods section for further details). Top row: B_2 inference results for simulations in which population size change occurred $N_{current}$ generations before sampling, where $N_{current}$ was the population size at time of sampling. Sampling occurred $75N_{ancestral}$ generations after the introduction of the balanced mutation, where $N_{ancestral}$ was the initial population size. Bottom row: *iHS* inference results for simulations in which the population size change occurs $0.01N_{ancestral}$ generations before sampling (the same time as the introduction of the balanced mutation). Inset plots show zoomed in ROC curves covering FPR values from 0 to 0.05. *iHS* and B_2 inference were performed at each SNP, and ROC curves were generated using 100bp windows. Exonic mutations were drawn from a DFE comprised of four fixed classes (following Johri et al. 2020), whose frequencies were denoted by f_i: f_0 with $0 \leq 2N_{ancestral}\, s < 1$ (*i.e.*, effectively neutral mutations), f_1 with $1 \leq 2N_{ancestral}\, s < 10$ (*i.e.*, weakly deleterious mutations), f_2 with $10 \leq 2N_{ancestral}\, s < 100$ (*i.e.,* moderately deleterious mutations), and f_3 with $100 \leq 2N_{ancestral}\, s$ (*i.e.*, strongly deleterious mutations), where s was the reduction in fitness of the mutant homozygote relative to wild-type. See Figure 2 for plots of DFEs.

S19

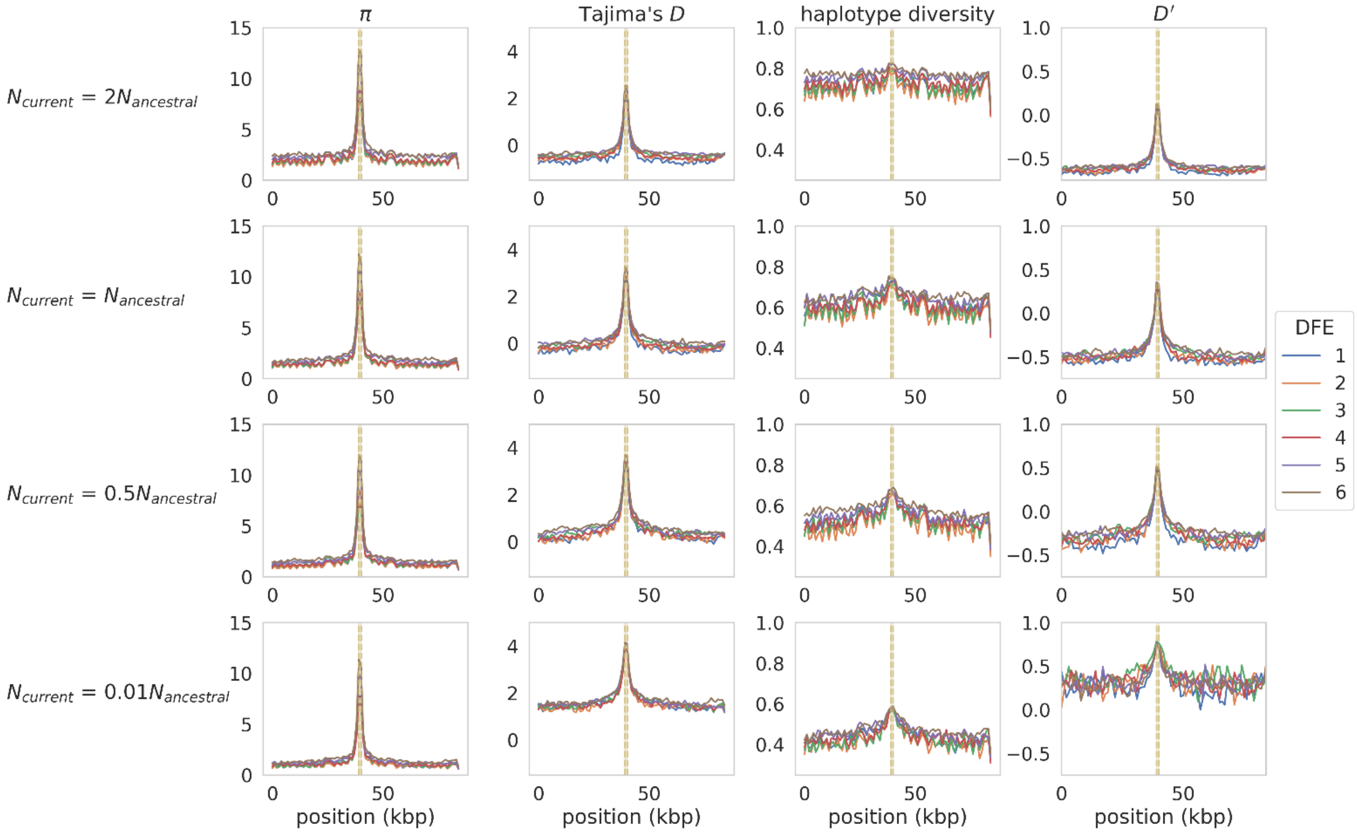

S19: Summary statistics for balancing selection simulations under 4 demographic histories across 6 DFEs, with **variable mutation and fixed recombination rates** across 200 simulated replicates. Population size change occurred $N_{current}$ generations before sampling, where $N_{current}$ was the population size at time of sampling. Sampling occurred $75N_{ancestral}$ generations after the introduction of the balanced mutation, where $N_{ancestral}$ was the initial population size. Exonic mutations were drawn from a DFE comprised of four fixed classes (following Johri et al. 2020), whose frequencies were denoted by f_i: f_0 with $0 \leq 2N_{ancestral}\, s < 1$ (*i.e.*, effectively neutral mutations), f_1 with $1 \leq 2N_{ancestral}\, s < 10$ (*i.e.*, weakly deleterious mutations), f_2 with $10 \leq 2N_{ancestral}\, s < 100$ (*i.e.*, moderately deleterious mutations), and f_3 with $100 \leq 2N_{ancestral}\, s$ (*i.e.*, strongly deleterious mutations), where s was the reduction in fitness of the mutant homozygote relative to wild-type. The shaded region represents windows in which the balanced mutation is segregating. Summary statistics were estimated using a window size of 2kb and a step size of 1kb. For variable rates, each 1kb region has a rate drawn from a uniform distribution such that each simulated replicate has the same mean rate as the fixed rate comparison (see Methods section for further details).

S20

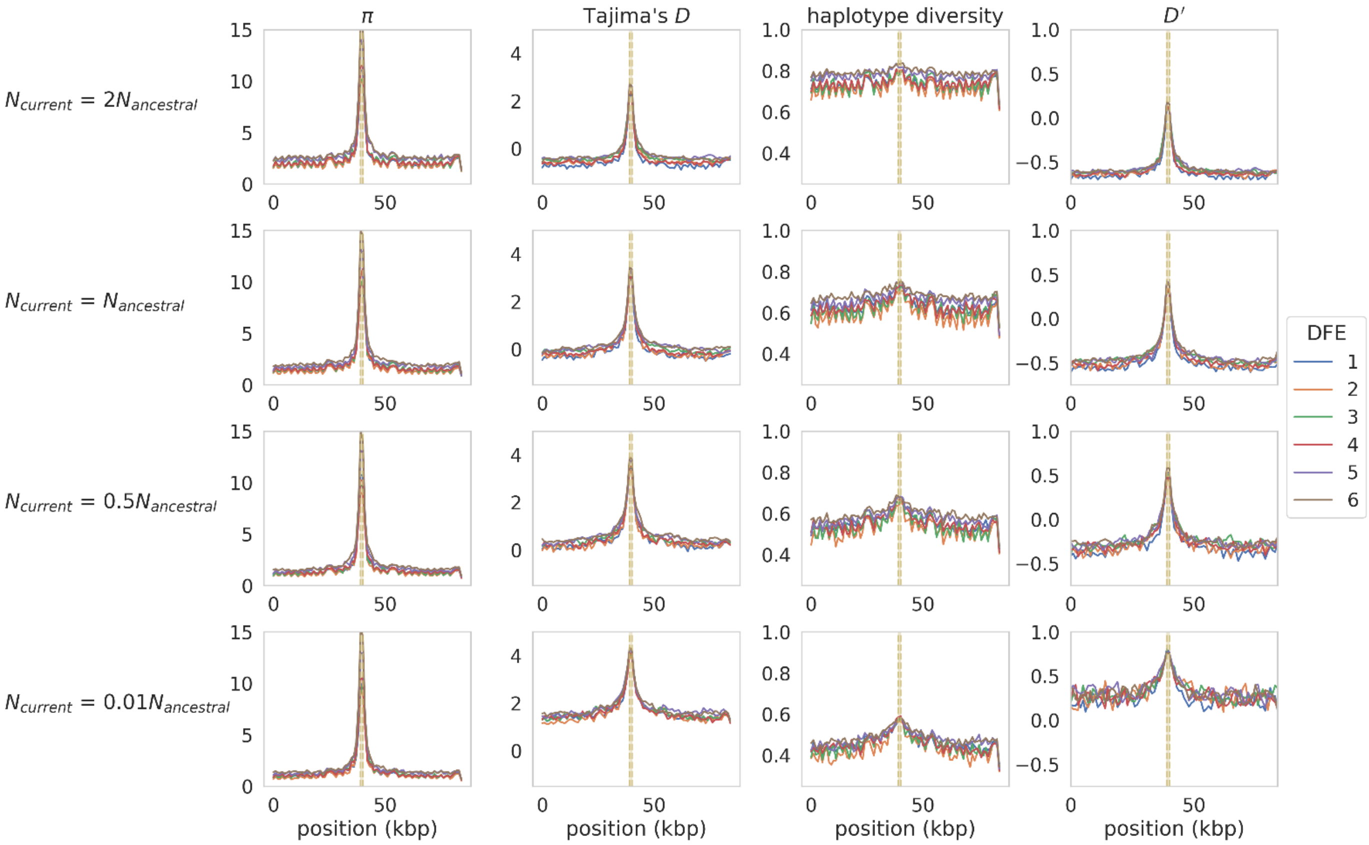

S20: Summary statistics for balancing selection simulations under 4 demographic histories across 6 DFEs, with **fixed mutation and variable recombination rates** across 200 simulated replicates. Population size change occurred $N_{current}$ generations before sampling, where $N_{current}$ was the population size at time of sampling. Sampling occurred $75N_{ancestral}$ generations after the introduction of the balanced mutation, where $N_{ancestral}$ was the initial population size. Exonic mutations were drawn from a DFE comprised of four fixed classes (following Johri et al. 2020), whose frequencies were denoted by f_i: f_0 with $0 \leq 2N_{ancestral}\,s < 1$ (*i.e.*, effectively neutral mutations), f_1 with $1 \leq 2N_{ancestral}\,s < 10$ (*i.e.*, weakly deleterious mutations), f_2 with $10 \leq 2N_{ancestral}\,s < 100$ (*i.e.*, moderately deleterious mutations), and f_3 with $100 \leq 2N_{ancestral}\,s$ (*i.e.*, strongly deleterious mutations), where s was the reduction in fitness of the mutant homozygote relative to wild-type. The shaded region represents windows in which the balanced mutation is segregating. Summary statistics were estimated using a window size of 2kb and a step size of 1kb. For variable rates, each 1kb region has a rate drawn from a uniform distribution such that each simulated replicate has the same mean rate as the fixed rate comparison (see Methods section for further details).

S21

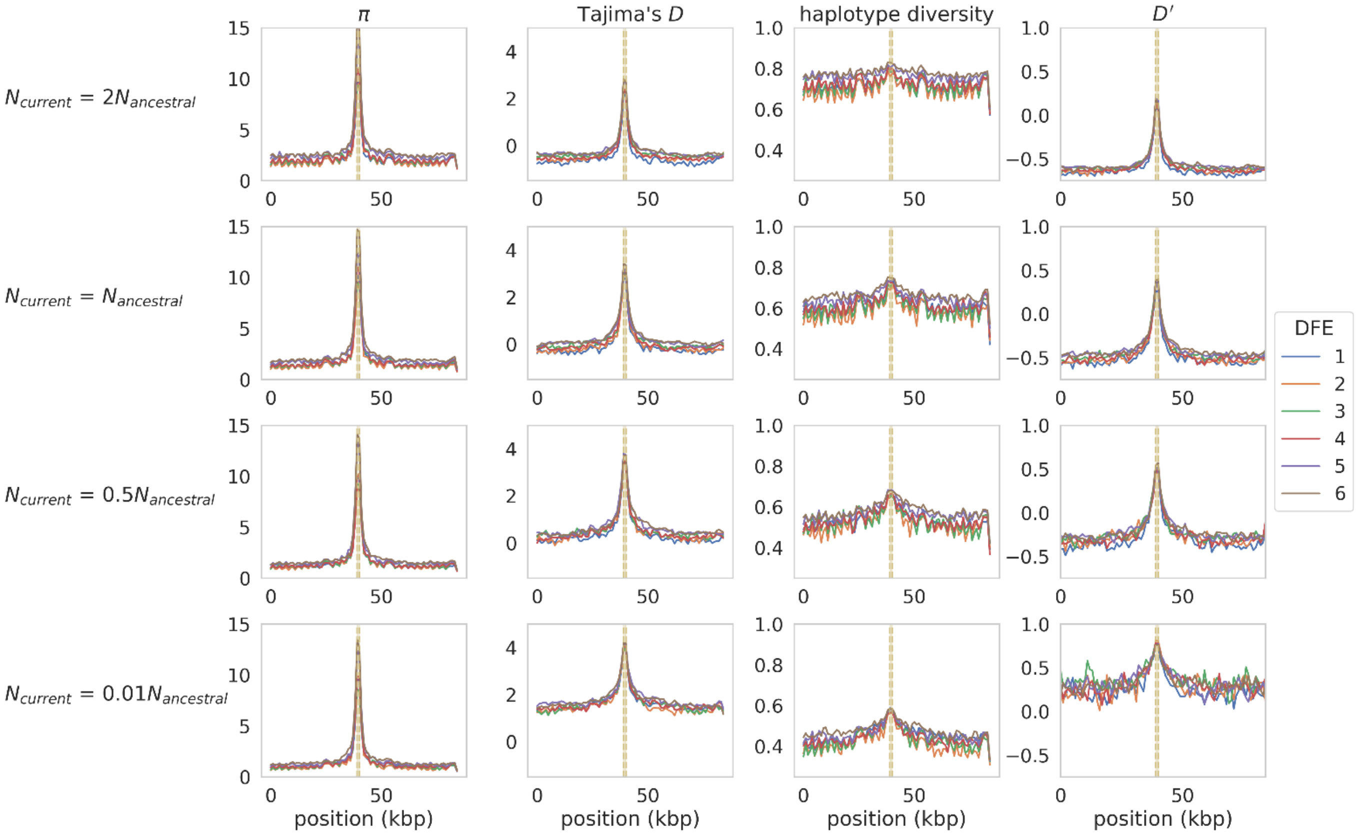

S21: Summary statistics for balancing selection simulations under 4 demographic histories across 6 DFEs, with **variable mutation and variable recombination rates** across 200 simulated replicates. Population size change occurred $N_{current}$ generations before sampling, where $N_{current}$ was the population size at time of sampling. Sampling occurred $75N_{ancestral}$ generations after the introduction of the balanced mutation, where $N_{ancestral}$ was the initial population size. Exonic mutations were drawn from a DFE comprised of four fixed classes (following Johri et al. 2020), whose frequencies were denoted by f_i: f_0 with $0 \leq 2N_{ancestral}\, s < 1$ (*i.e.*, effectively neutral mutations), f_1 with $1 \leq 2N_{ancestral}\, s < 10$ (*i.e.*, weakly deleterious mutations), f_2 with $10 \leq 2N_{ancestral}\, s < 100$ (*i.e.*, moderately deleterious mutations), and f_3 with $100 \leq 2N_{ancestral}\, s$ (*i.e.*, strongly deleterious mutations), where s was the reduction in fitness of the mutant homozygote relative to wild-type. The shaded region represents windows in which the balanced mutation is segregating. Summary statistics were estimated using a window size of 2kb and a step size of 1kb. For variable rates, each 1kb region has a rate drawn from a uniform distribution such that each simulated replicate has the same mean rate as the fixed rate comparison (see Methods section for further details).

S22

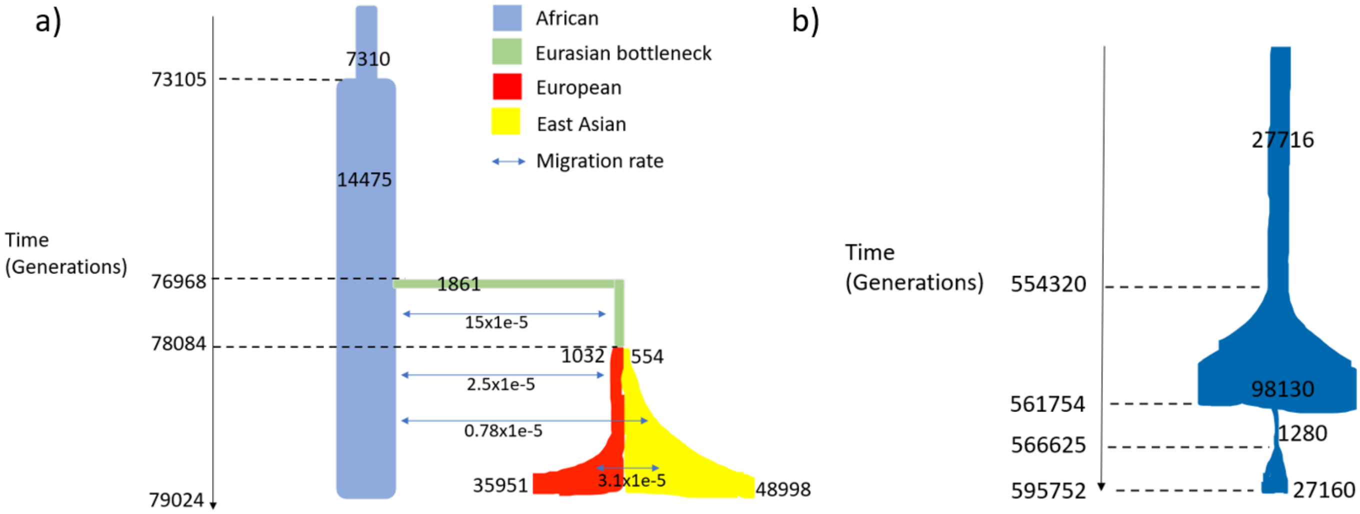

S22: Schematics of a) the Gravel et al. (2011) Out-Of-Africa model of human demography and b) the modified Hu et al. (2023) model of African demographic history.

S23

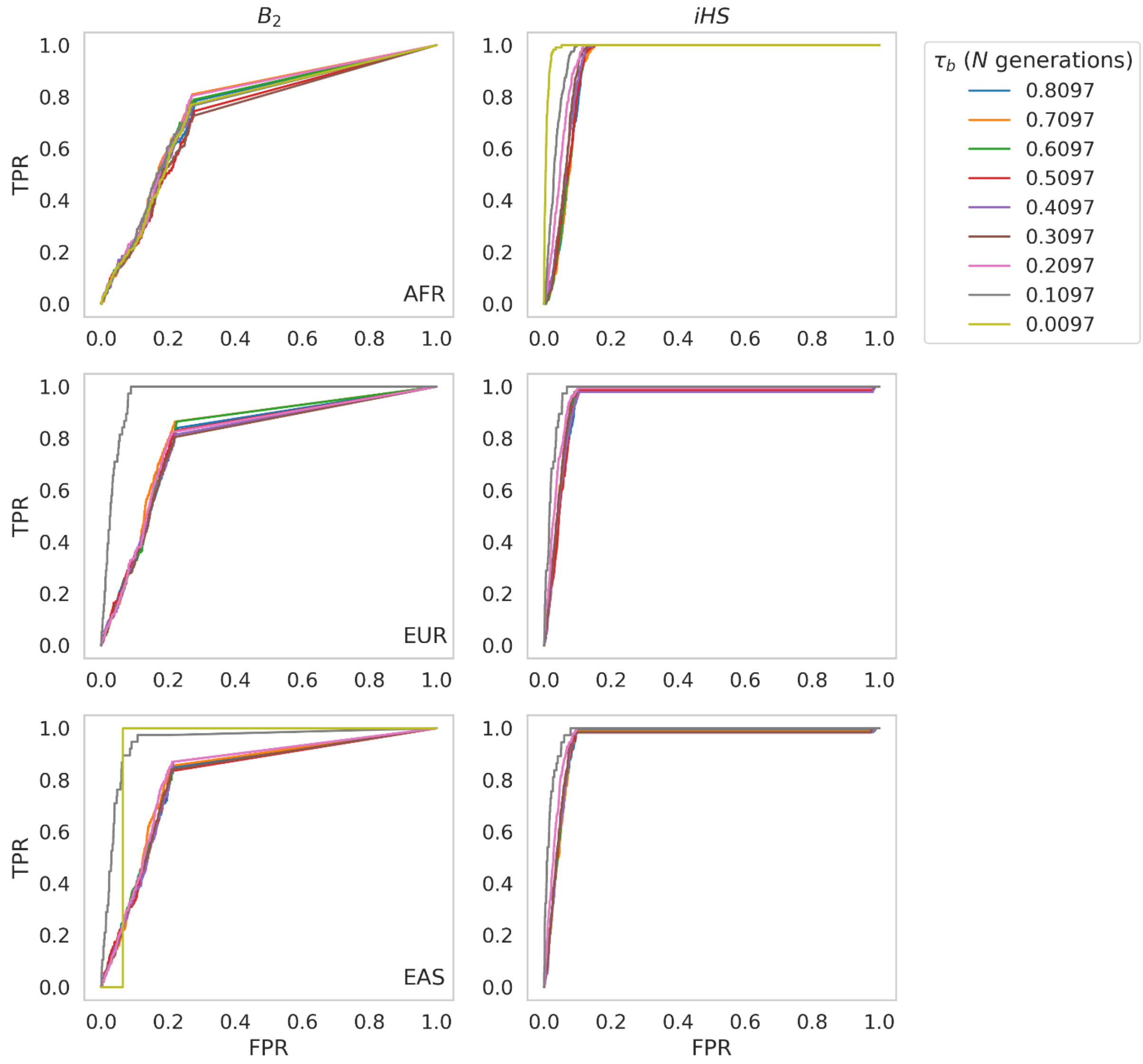

S23: ROC curves presenting the change in true-positive rate (TPR) as the false-positive rate (FPR) increases, for balancing selection inference under the Gravel et al. (2011) model of human demography, with fixed recombination and fixed mutation rates across 200 simulated replicates, using the B_2 method and the *iHS* statistic. **The balanced mutation was introduced in the African population** (see Methods section for further details). The balanced mutation was introduced at a number of different time points, τ_b (time since the introduction of the balanced mutation). Each row is for a different sampled population (AFR: African; EUR: European; EAS: East Asian). *iHS* and B_2 inference were performed at each SNP, and ROC curves were generated using 100bp windows. Exonic mutations were drawn from a DFE inferred from human data, comprised of four fixed classes (Johri et al. 2023), whose frequencies were denoted by f_i: f_0 with $0 \leq 2N_{ancestral}\, s < 1$ (*i.e.*, effectively neutral mutations), f_1 with $1 \leq 2N_{ancestral}\, s < 10$ (*i.e.*, weakly deleterious mutations), f_2 with $10 \leq 2N_{ancestral}\, s < 100$ (*i.e.*, moderately deleterious mutations), and f_3 with $100 \leq 2N_{ancestral}\, s$ (*i.e.*, strongly deleterious mutations), where *s* was the reduction in fitness of the mutant homozygote relative to wild-type.

S24

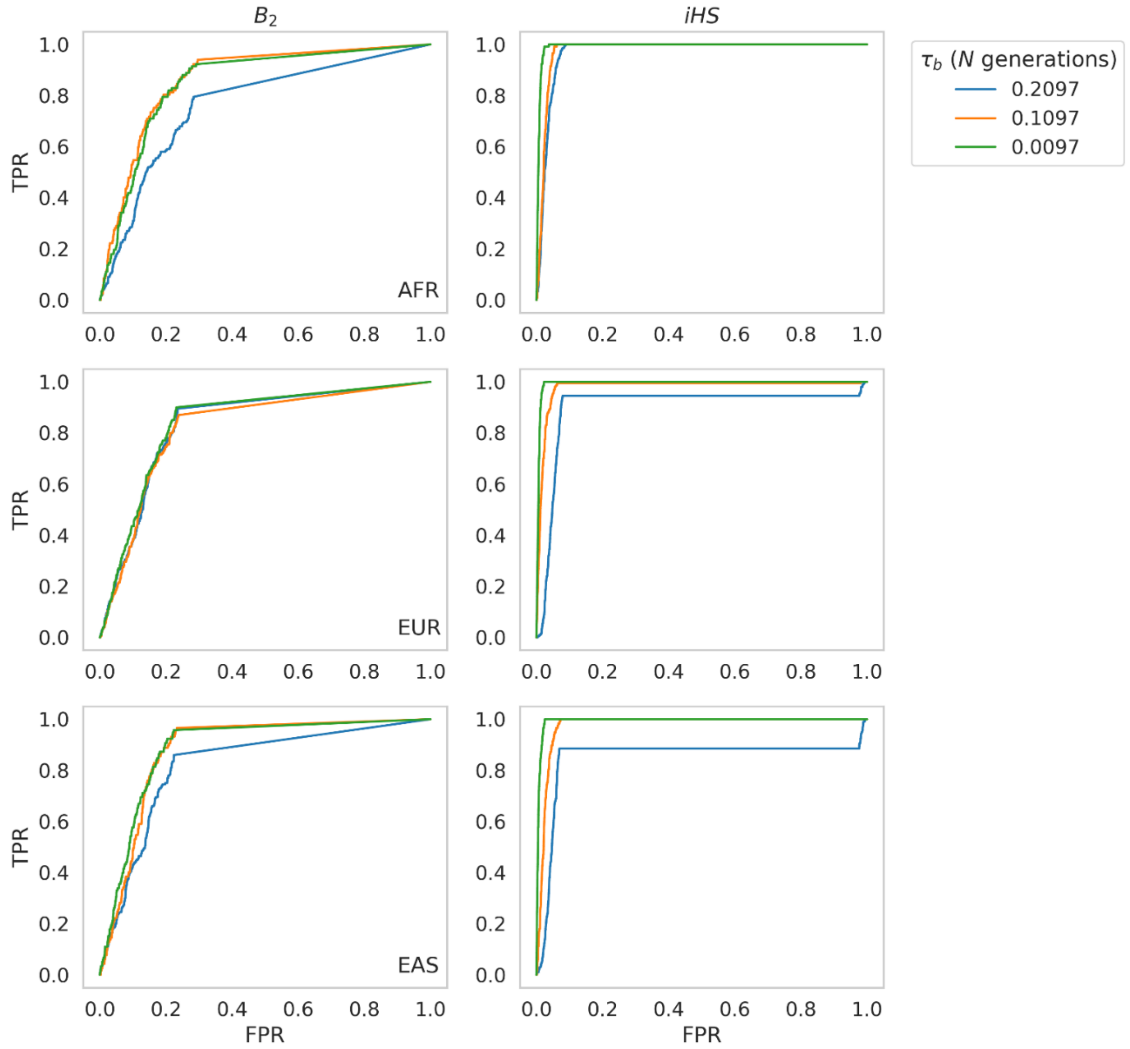

S24: ROC curves presenting the change in true-positive rate (TPR) as the false-positive rate (FPR) increases, for balancing selection inference under the Gravel et al. (2011) model of human demography, with fixed recombination and fixed mutation rates across 200 simulated replicates, using the B_2 method and the *iHS* statistic. **The balanced mutation was introduced in the European population** (see Methods section for further details). The balanced mutation was introduced at a number of different time points, τ_b (time since the introduction of the balanced mutation). Each row is for a different sampled population (AFR: African; EUR: European; EAS: East Asian). *iHS* and B_2 inference were performed at each SNP, and ROC curves were generated using 100bp windows. Exonic mutations were drawn from a DFE inferred from human data, comprised of four fixed classes (Johri et al. 2023), whose frequencies were denoted by f_i: f_0 with $0 \leq 2N_{ancestral}\, s < 1$ (*i.e.*, effectively neutral mutations), f_1 with $1 \leq 2N_{ancestral}\, s < 10$ (*i.e.*, weakly deleterious mutations), f_2 with $10 \leq 2N_{ancestral}\, s < 100$ (*i.e.*, moderately deleterious mutations), and f_3 with $100 \leq 2N_{ancestral}\, s$ (*i.e.*, strongly deleterious mutations), where *s* was the reduction in fitness of the mutant homozygote relative to wild-type.

S25

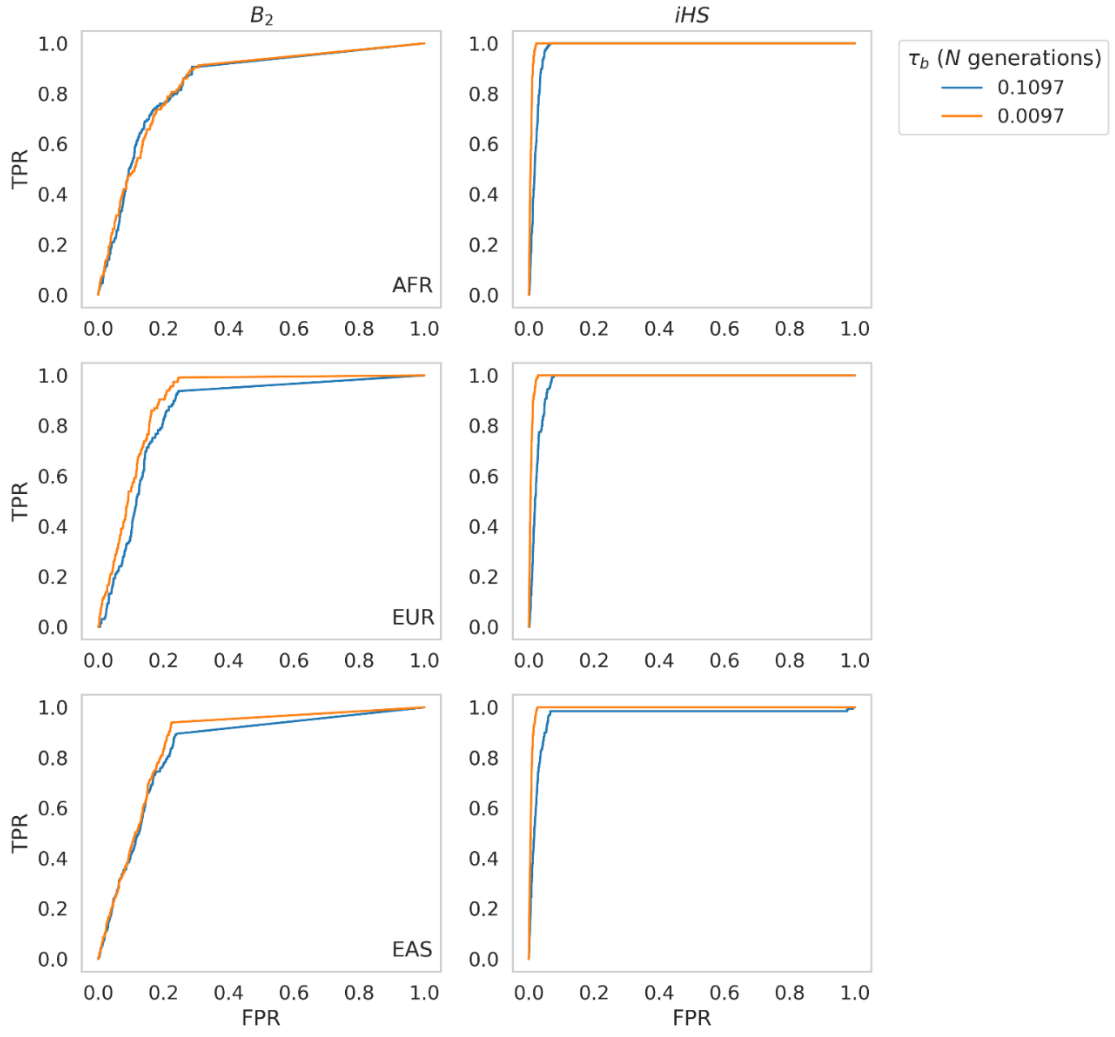

S25: ROC curves presenting the change in true-positive rate (TPR) as the false-positive rate (FPR) increases, for balancing selection inference under the Gravel et al. (2011) model of human demography, with fixed recombination and fixed mutation rates across 200 simulated replicates, using the B_2 method and the *iHS* statistic. **The balanced mutation was introduced in the East Asian population** (see Methods section for further details). The balanced mutation was introduced at a number of different time points, τ_b (time since the introduction of the balanced mutation). Each row is for a different sampled population (AFR: African; EUR: European; EAS: East Asian). *iHS* and B_2 inference were performed at each SNP, and ROC curves were generated using 100bp windows. Exonic mutations were drawn from a DFE inferred from human data, comprised of four fixed classes (Johri et al. 2023), whose frequencies were denoted by f_i: f_0 with $0 \leq 2N_{ancestral}\, s < 1$ (*i.e.*, effectively neutral mutations), f_1 with $1 \leq 2N_{ancestral}\, s < 10$ (*i.e.*, weakly deleterious mutations), f_2 with $10 \leq 2N_{ancestral}\, s < 100$ (*i.e.*, moderately deleterious mutations), and f_3 with $100 \leq 2N_{ancestral}\, s$ (*i.e.*, strongly deleterious mutations), where *s* was the reduction in fitness of the mutant homozygote relative to wild-type.

S26

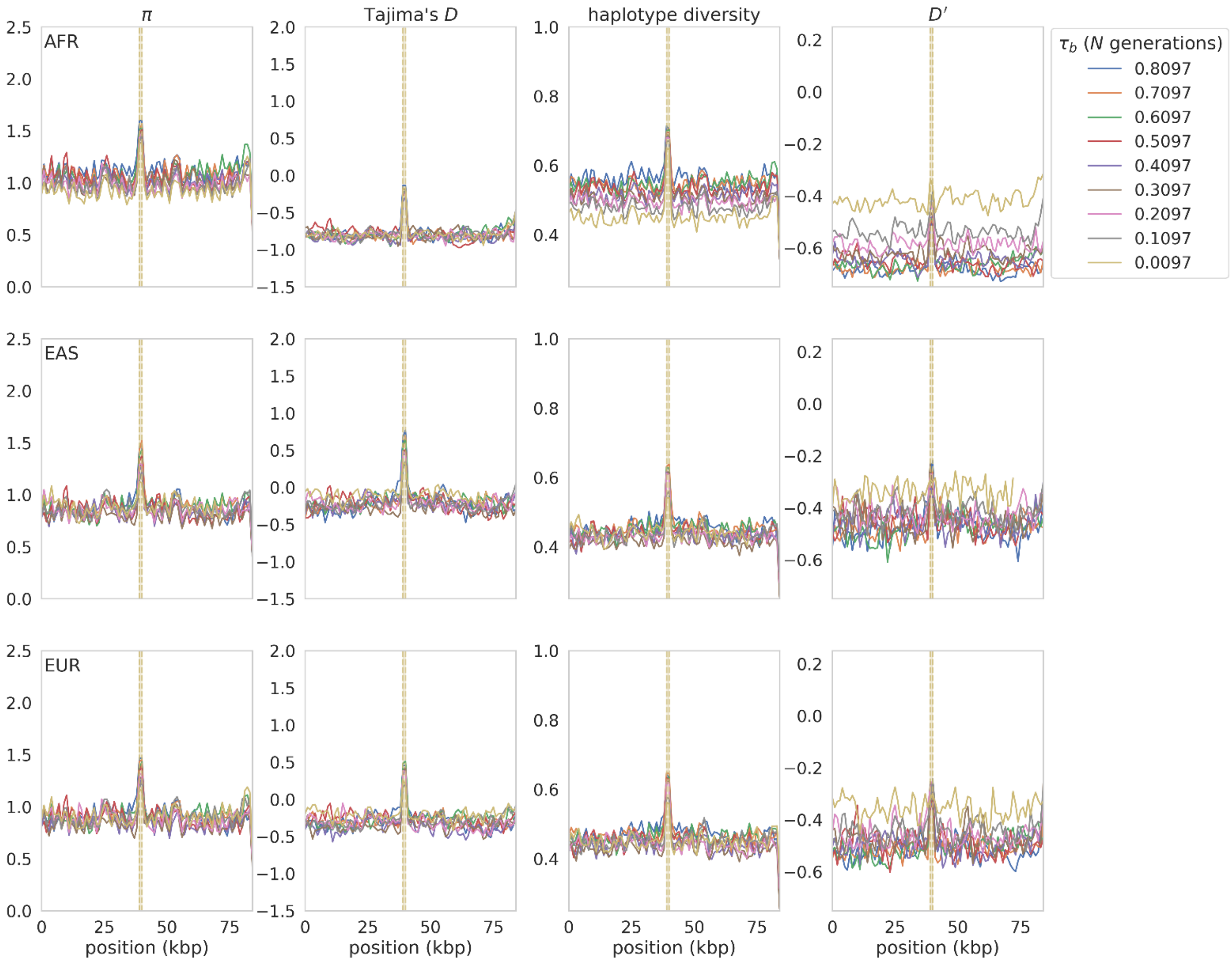

S26: Summary statistics for balancing selection simulations under the Gravel et al. (2011) model of human demography, with fixed recombination and fixed mutation rates across 200 simulated replicates. Each row is for a different sampled population, in which the balanced mutation was introduced (AFR: African; EUR: European; EAS: East Asian). The balanced mutation was introduced at a number of different time points, τ_b (time since the introduction of the balanced mutation). Exonic mutations were drawn from a DFE inferred from human data, comprised of four fixed classes (Johri et al. 2023), whose frequencies were denoted by f_i: f_0 with $0 \le 2N_{ancestral}\, s < 1$ (*i.e.*, effectively neutral mutations), f_1 with $1 \le 2N_{ancestral}\, s < 10$ (*i.e.*, weakly deleterious mutations), f_2 with $10 \le 2N_{ancestral}\, s < 100$ (*i.e.*, moderately deleterious mutations), and f_3 with $100 \le 2N_{ancestral}\, s$ (*i.e.*, strongly deleterious mutations), where *s* was the reduction in fitness of the mutant homozygote relative to wild-type. The shaded region represents windows in which the balanced mutation is segregating. Summary statistics were estimated using a window size of 2kb and a step size of 1kb.

S27

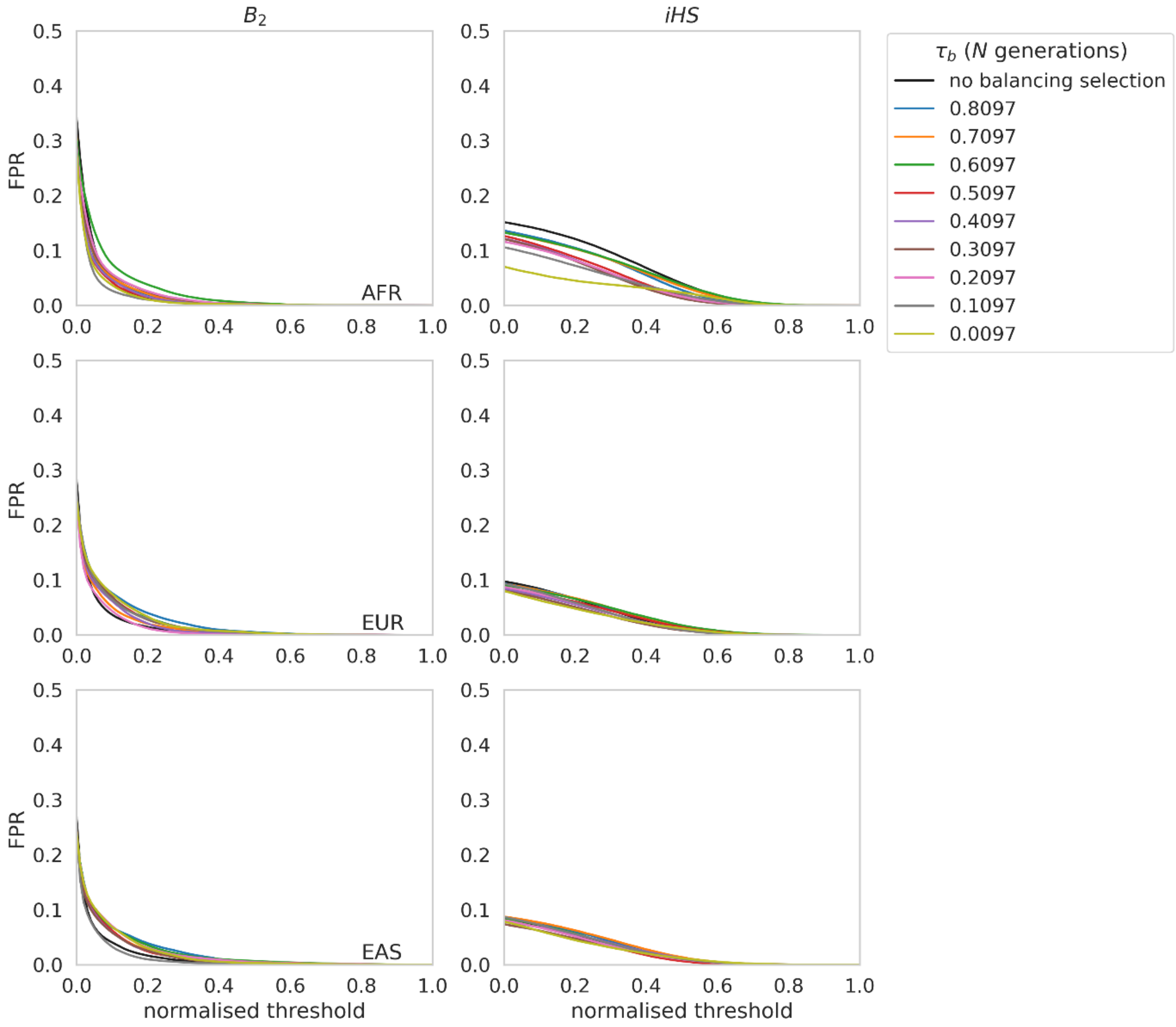

S27: False positive rates plotted against the normalised threshold, for balancing selection inference under the Gravel et al. (2011) model of human demography, with fixed recombination and fixed mutation rates across 200 simulated replicates, using the B_2 method (left) and the *iHS* statistic (right). **The balanced mutation was introduced in the African population** (see Methods section for further details). The balanced mutation was introduced at a number of different time points, τ_b (time since the introduction of the balanced mutation). The black line presents a scenario with no balancing selection. Because thresholds will depend on the minimum and maximum CLR or *iHS* values, these were normalised to between 0 and 1. Each row represents a different sampled population (AFR: African; EUR: European; EAS: East Asian). *iHS* and B_2 inference were performed at each SNP, and FPR plots were generated using 100bp windows. Exonic mutations were drawn from a DFE inferred from human data, comprised of four fixed classes (Johri et al. 2023), whose frequencies were denoted by f_i: f_0 with $0 \leq 2N_{ancestral}\, s < 1$ (*i.e.*, effectively neutral mutations), f_1 with $1 \leq 2N_{ancestral}\, s < 10$ (*i.e.*, weakly deleterious mutations), f_2 with $10 \leq 2N_{ancestral}\, s < 100$ (*i.e.*, moderately deleterious mutations), and f_3 with $100 \leq 2N_{ancestral}\, s$ (*i.e.*, strongly deleterious mutations), where *s* was the reduction in fitness of the mutant homozygote relative to wild-type.

S28

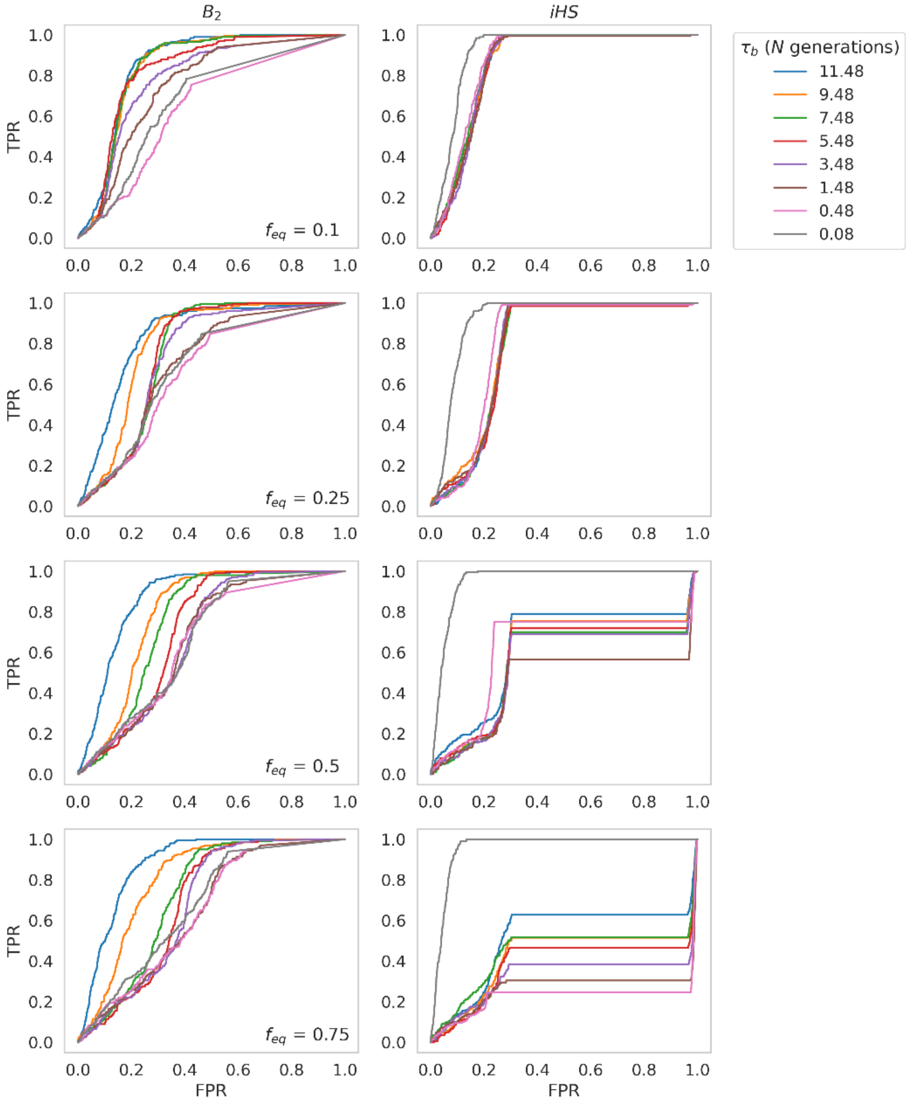

S28: ROC curves presenting the change in true-positive rate (TPR) as the false-positive rate (FPR) increases, for balancing selection inference under the Hu et al. (2023) model of human demography, with fixed recombination and fixed mutation rates across 200 simulated replicates, using the B_2 method (left) and the *iHS* statistic (right). The balanced mutation was introduced at a number of different time points, τ_b (time since the introduction of the balanced mutation). Each row is for a different sampled population equilibrium frequency (f_{eq}) of the balanced mutation. *iHS* and B_2 inference were performed at each SNP, and ROC curves were generated using 100bp windows. Exonic mutations were drawn from a DFE inferred from human data, comprised of four fixed classes (Johri et al. 2023), whose frequencies were denoted by f_i: f_0 with $0 \leq 2N_{ancestral}\, s < 1$ (*i.e.*, effectively neutral mutations), f_1 with $1 \leq 2N_{ancestral}\, s < 10$ (*i.e.*, weakly deleterious mutations), f_2 with $10 \leq 2N_{ancestral}\, s < 100$ (*i.e.*, moderately deleterious mutations), and f_3 with $100 \leq 2N_{ancestral}\, s$ (*i.e.*, strongly deleterious mutations), where *s* was the reduction in fitness of the mutant homozygote relative to wild-type.

S29

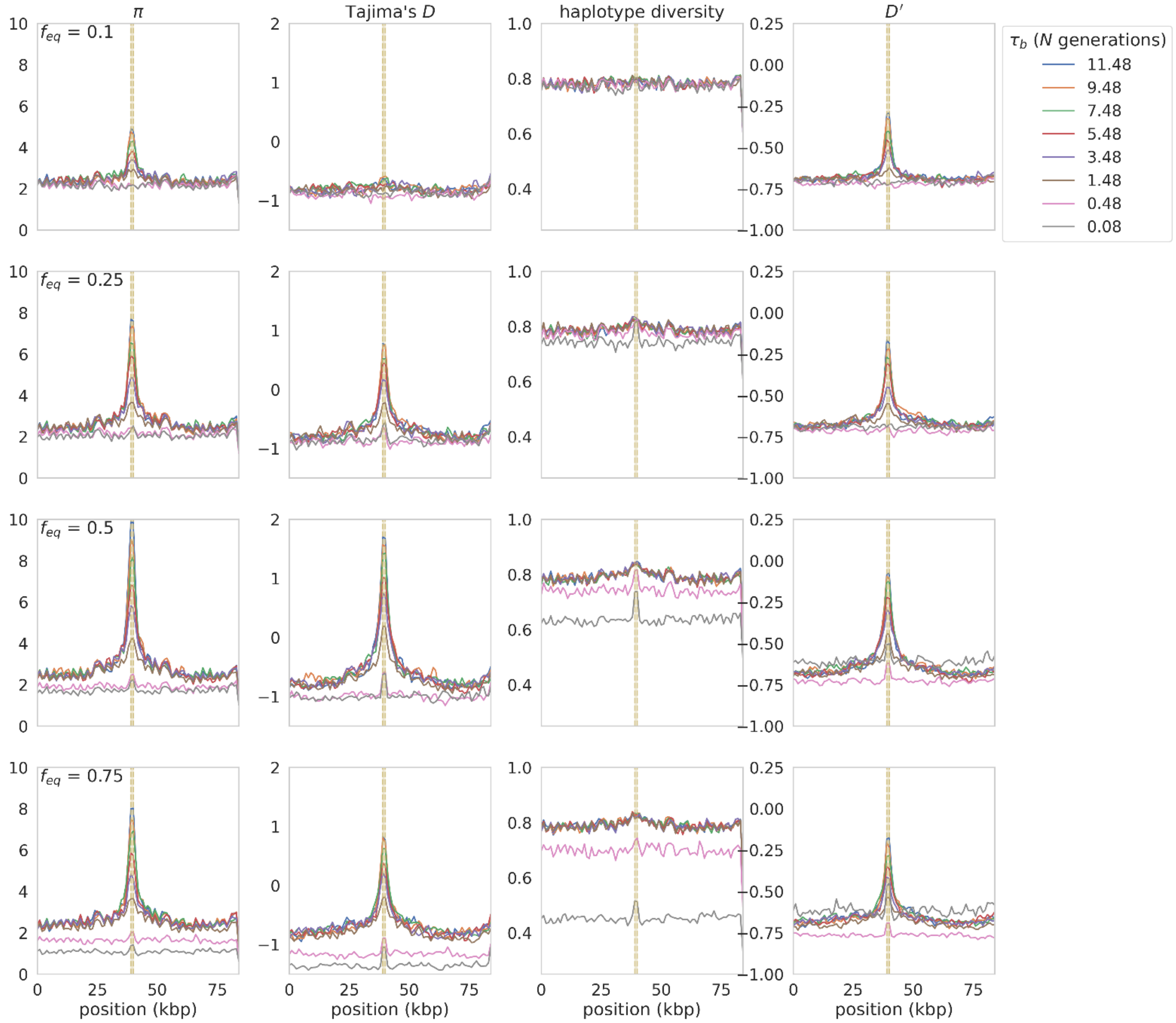

S29: Summary statistics for balancing selection simulations under the Hu et al. (2023) model of human demography, with fixed recombination and fixed mutation rates across 200 simulated replicates. Each row is for a different sampled population equilibrium frequency (f_{eq}) of the balanced mutation. The balanced mutation was introduced at a number of different time points, τ_b (time since the introduction of the balanced mutation). Exonic mutations were drawn from a DFE inferred from human data, comprised of four fixed classes (Johri et al. 2023), whose frequencies were denoted by f_i: f_0 with $0 \leq 2N_{ancestral}\, s < 1$ (*i.e.*, effectively neutral mutations), f_1 with $1 \leq 2N_{ancestral}\, s < 10$ (*i.e.*, weakly deleterious mutations), f_2 with $10 \leq 2N_{ancestral}\, s < 100$ (*i.e.*, moderately deleterious mutations), and f_3 with $100 \leq 2N_{ancestral}\, s$ (*i.e.*, strongly deleterious mutations), where *s* was the reduction in fitness of the mutant homozygote relative to wild-type. The shaded region represents windows in which the balanced mutation is segregating. Summary statistics were estimated using a window size of 2kb and a step size of 1kb.
